# The role of interferons in ovarian cancer progression: Hinderer or promoter?

**DOI:** 10.3389/fimmu.2022.1087620

**Published:** 2022-12-21

**Authors:** Taiqing Liu, Yinqi Li, Xiaoyu Wang, Xiaodong Yang, Yunhai Fu, Yeteng Zheng, Hanlin Gong, Zhiyao He

**Affiliations:** ^1^ Department of Pharmacy, State Key Laboratory of Biotherapy and Cancer Center, West China Hospital, Sichuan University, Chengdu, China; ^2^ Department of Integrated Traditional Chinese and Western Medicine, West China Hospital, Sichuan University, Chengdu, China; ^3^ Key Laboratory of Drug-Targeting and Drug Delivery System of the Education Ministry, Sichuan Engineering Laboratory for Plant-Sourced Drug and Sichuan Research Center for Drug Precision Industrial Technology, West China School of Pharmacy, Sichuan University, Chengdu, China

**Keywords:** interferons, ovarian cancer, tumor microenvironment, immune cell, immunotherapy, biotherapy

## Abstract

Ovarian cancer (OC) is a common gynecologic malignancy with poor prognosis and high mortality. Changes in the OC microenvironment are closely related to the genesis, invasion, metastasis, recurrence, and drug-resistance. The OC microenvironment is regulated by Interferons (IFNs) known as a type of important cytokines. IFNs have a bidirectional regulation for OC cells growth and survival. Meanwhile, IFNs positively regulate the recruitment, differentiation and activation of immune cells. This review summarizes the secretion and the role of IFNs. In particular, we mainly elucidate the actions played by IFNs in various types of therapy. IFNs assist radiotherapy, targeted therapy, immunotherapy and biotherapy for OC, except for some IFN pathways that may cause chemo-resistance. In addition, we present some advances in OC treatment with the help of IFN pathways. IFNs have the ability to powerfully modulate the tumor microenvironment and can potentially provide new combination strategies for OC treatment.

## Introduction

Ovarian cancer (OC) has an insidious onset and a bad prognosis. And OC screening is not effective in reducing mortality (1). Nearly half of patients are diagnosed at stage III, when survival rates sharply decrease. The current main treatments are chemotherapy and surgery. Patients initially respond to treatment, but most patients ultimately relapse with resistant OC. Worse still, the global number of incident cases and deaths of OC increased as data showed from 1990 to 2019 (2). Therefore, new treatments or effective combinations need to be further investigated.

Findings suggest that the tumor microenvironment (TME) plays an important role in the development of OC. And an increasing amount of attention has been given to TME as a therapeutic potential in recent years. Interferons (IFNs) are an important class of pleiotropic cytokines in the OC microenvironment and are divided into three subtypes. In OC, one studies type-I-IFN (IFN-I) and type-II-IFN (IFN-II). Most cells in OC can secrete them, and various types of immune cells and cancer cells make a larger contribution. Accordingly, IFNs can regulate almost all cells in the OC microenvironment. IFNs directly affect not only tumor cell production, cell cycle, stemness property, migration, and drug resistance, but also immune cell recruitment, differentiation, activation, and immune activity by regulating cellular gene expression. Ultimately, IFNs have a huge impact on tumor progression. IFN treatment has a good clinical response in hematologic malignancies (hairy cell leukemia and chronic myeloid leukemia) and certain solid tumors (melanoma, renal cancer and AIDS-related Kaposi’s sarcoma) through itself or as part of combination treatment (3–7). Although IFNs have received long-standing concerns in the study and treatment of OC, clinical studies of IFNs in OC have not yet achieved breakthroughs. A summary of studies on the mechanisms of different OC treatments revealed that many treatments need to function through IFNs, and perturbation of IFN signalling pathway could lead to the inability of some drugs. Therefore, make good use of IFN responses are beneficial to the treatment of OC. With the deepening of immunotherapy in recent years, the cooperation between IFNs and immunotherapy seemed to be effective. This review summarizes many associations between OC and IFNs, expecting to provide a reference for improving the cold OC microenvironment.

## Secretion and regulation of IFNs in the microenvironment of OC

**Figure 1:**
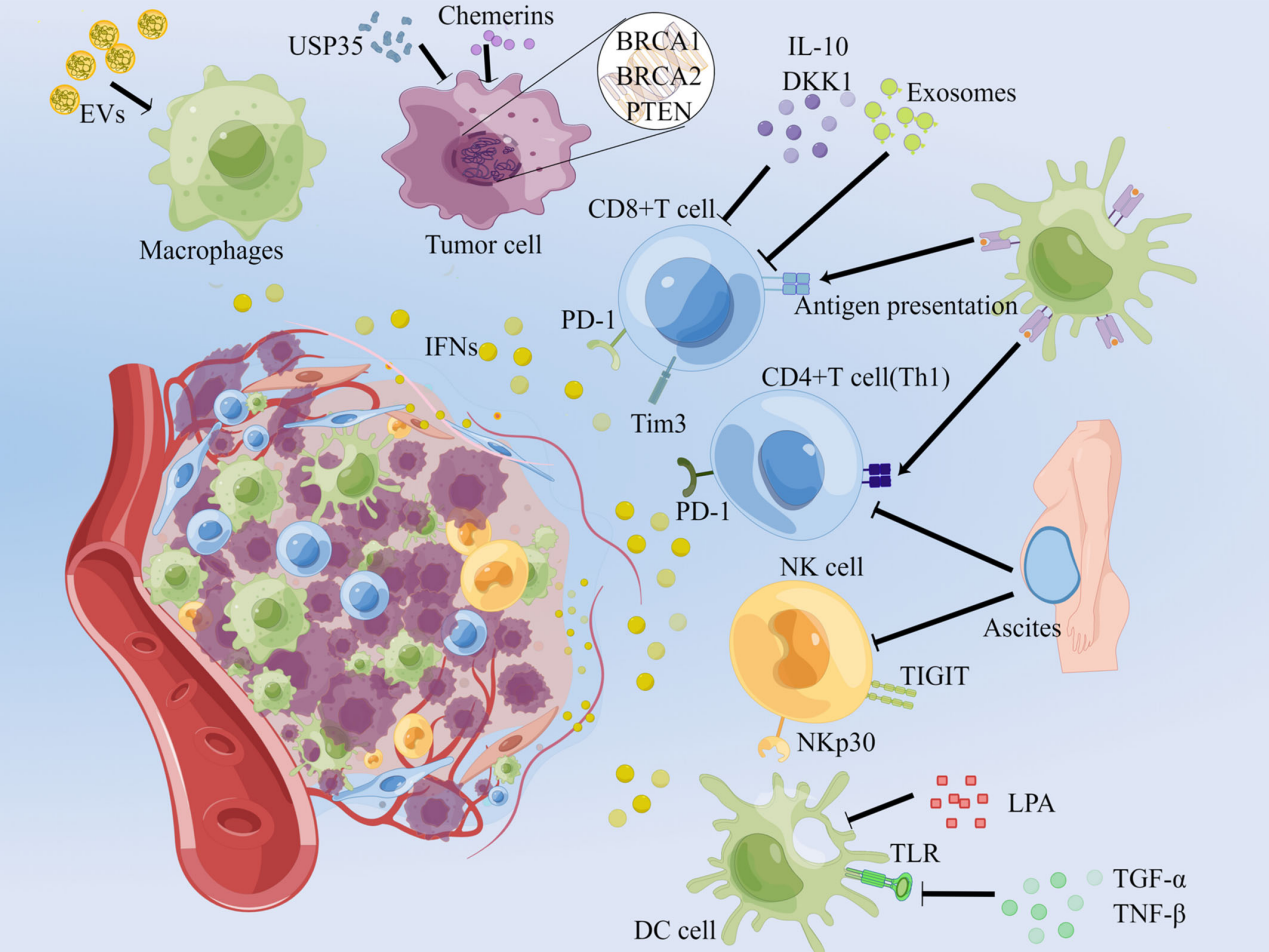
Secretion and regulation of IFNs in the OC microenvironment. Normal arrows represent the up-regulators of IFNs secretion, and T-shaped arrows imply barriers. In the OC microenvironment, EVs enriched in nucleic acids stimulate macrophages to secrete IFNs; unstable genes in OC cells result in the secretion of IFNs; PD-1^+^ Tim3^+^ tumor-infiltrating CD8^+^ T cells are able to continuously secrete IFN-γ; blocking the PD-1 of CD4^+^ T, CD8^+^ T cells and TIGIT of NK cells can reduce IFN-γ; and promoting the activating receptor NKp30 plays the inverse effect. EVs: extracellular vesicles, DKK1: dickkopf-related protein 1, LPA: lysophosphatidic acid. This picture is drawn with the help of Figdraw.

The TME in OC is extremely complex that various types of cytokines and cells exist. The main sources of IFNs are immune cells in the microenvironment. For example, pDCs (plasmacytoid dendritic cells) are the prime origin of IFN-I, CD8^+^ T cells, NK cells and Th1 CD4^+^ T cells are the main source of IFN-γ. And macrophages, cancer cells and etc. also secrete IFNs under the regulation of the microenvironment (8, 9) (**Figure 1**).

### Tumor-infiltrating lymphocytes

Tumor-infiltrating lymphocytes (TILs), such as NK cells, CD4^+^ and CD8^+^ T cells play important roles in the OC microenvironment by secreting IFNs with immunity. Ascites contains a large number of CD4^+^ and CD8^+^ T cells, both of which can produce large amounts of IFN-γ. Compared to T cells in the blood of healthy body, however, T cells secretion ability of IFN-γ was relatively lower in blood, ascites and tumor tissue of patients with epithelial ovarian cancer (EOC) (10). Normally, CD8^+^ T cells eradicate tumor cells by secreting granzyme B, TNF and IFN-γ after TCR attachment. Yet these TCRs in TMA cannot be awakened and recognized causing decrease in IFNs and development of OC (11, 12). Percentage of γδ T cells (innate lymphocytes with unbound MHC) in OC tissues was significantly higher than the cells in normal ovarian tissues. In contrast, patients had lesser levels of IFN-γ secretion by γδ T cells in both peripheral blood and cancer tissues compared with the healthy and benign OC patients (13). Furthermore, when CD8^+^ T cells were exhausted due to continuous exposure to tumor antigens in the OC microenvironment, they would express the immune checkpoint molecules PD-1, and Tim3. Cells with such characteristics had the ability to consistently produce IFN-γ (14). Th1 CD4^+^ T cells produced high levels of IFN-γ in response to antigen stimulation (15). NK cells from OC ascites had the same ability to produce IFN-γ as healthy donor peripheral blood-NK cells. TILs would tend to secrete a great deal of IFNs to exert their antitumor effects under physiological conditions. Nevertheless, some external disturbances would modulate their secretory effects. Malignant ascites in OC patients inhibited glucose uptake and caused defective N-linked protein glycosylation in T cells, which triggered IRE1α-XBP1 activation that inhibited mitochondrial activity and IFN-γ expression (16). CD8^+^ T cells co-cultured with B cells in ascites exhibited significant suppression of IFN-γ production, which was later found to be associated with IL-10 expression and low CD80/CD86. When IL-10 depletion was stimulated with CD28, IFN-γ secretion would be upregulated (17). This phenomenon was also present in other cancers in which IL-10 affected the signaling pathway and expression of IFN-γ (18). Mutation in the Wnt pathway was a hallmark of the endometrioid and clear cell subtypes of EOC. Dickkopf-related protein 1 (DKK1) overexpression associated with Wnt mutation decreased IFN-γ secretion from CD8^+^ T cells (19). There was a decreasing expression of the NK cell activating receptor NKp30 in 50% peritoneal fluid of patients with serous tissue type OC. The fewer NKp30 may be associated with B7-H6 and cause a decrease in IFN-γ (20). TIGIT is an inhibitory receptor on the surface of NK cells. when it was blocked, the ability of NK cells’ secretion of IFN-γ to would be increased in OC (21). CD4^+^ and CD8^+^ T cells isolated from OC patients were subjected to a significant increase in IFN-γ after the use of PD-1 blocking antibodies (22). Overexpression of Pyruvate dehydrogenase kinase 1 (PDK1) in OC cells impaired IFN-γ secretion in CD8^+^ T cells by upregulating PD-L1, and an increase in intra-tumor of IFN-γ was observed with DCA (a PDK inhibitor) and anti-PD-L1 antibodies (23). Exosomes in the ascites of OC patients had an inhibition on IFN-γ secretion for CD4^+^ and CD8^+^ T cells, but the suppression can only be maintained within 24-48 hours after disconnection from exosomes (24). Although tumor antigens can stimulate TILs to secrete IFNs, the ability of secretion IFNs for TILs in OC tissues was impaired in general, which may be an important reason for the suppressive immune microenvironment in OC.

### Antigen presenting cells

Antigen-presenting cells significantly contribute to IFNs production, especially dendritic cells (DCs). PDCs produce large amounts of IFN-I upon stimulation. TLR7 and TLR9 of pDCs were activated to produce IFN-α for tumor-killing (25, 26). Nevertheless, the IFNs secretion function of pDCs is repressed under some conditions. The ligands of the TCR9 and TCR9 were blocked in OC by the action of soluble factors TGF-β and TNF-α cooperation, causing a decrease of IFNs (27). In addition, recent studies have found that lysophosphatidic acid (LPA) was abundant in malignant ascites. It was derived from ATX, which was released by OC cells. LPA triggered the biosynthesis of PGE2 in multiple subtypes of DCs, which inhibited IFN-I signal transduction through the involvement of autocrine EP4. This signal down-regulated multiple IFN stimulated genes (ISGs), which reduced activation and infiltration of CD8^+^ T cells and NK cells (28). Aberrant epigenetic modifications of TP53 in OC can generate aberrant repeat genes. EVs (extracellular vesicles) can enrich these repeat RNAs, which were then sensed by pattern recognition receptors and induced IFN-I responses in human primitive monocyte and macrophage cell lines (29).

### OC cells

Cancer cells are usually accompanied by genetic instability, which causes the production and accumulation of abnormal RNA or DNA, and ultimately induces IFN responses. BRCA mutations are common in OC. It is found that BRCA1 loss allowed OC cells to exhibit a cell-autonomous inflammatory state leading to chromatin reorganization and transcriptional reprogramming. By increasing the sensitivity of the dsDNA sensing pathway and enhancing the supply of cytoplasmic dsDNA to STING (stimulator of interferon genes), DNA sensing and inflammatory (DS/IFN) pathway was overexpressed, thereby causing IFN responses (30, 31). Tumor-prone cells carrying BRCA2 inactivation underwent loss of chromosomal integrity and accumulated cell membrane DNA in the form of micronuclei. Micronucleus bound DNA sensor cGAS and activated the IFN responses (32). In addition to BRCA mutations, PTEN mutation also affected IFN signaling. PTEN deficiency failed to activate the IFN signaling pathway, thus promoting a tumor immunosuppressive microenvironment (33). Furthermore, Jiawei Zhang et al. found that the ubiquitinase USP35 was upregulated in OC tissues, and the upregulation may inhibit IFN-I expression in cancer cells through the STING-TBK1-IRF3 pathway (34). Chemerin is a pleiotropic adipokine that has an important role in the immune system. It was found that co-culture of Chemerin and OC cell lines increased the level of IFN-α approximately fourfold in the culture medium, thereby activating IFN-α responsive genes, such as IFI27, OAS1 and IFIT1, IFI44L, its upstream regulator IRF9 and etc. (35). Although OC cells induce IFN responses and promote immune responses due to their “self-deficiency”, the IFN responses are almost ineffective. Immune cells are damaged by OC TAM, which makes it difficult for these immune cells to survive and respond to the stimulation of IFNs. In summary, IFNs can be expressed by a variety of cells in the OC microenvironment. Apart from cells mentioned above, tumor-associated fibroblasts and endothelial cells also express IFN-I. The relative contribution of each cell to total IFNs levels may depend on the quantity and quality of each cell type within the tumor and is regulated in multiple layers within the OC microenvironment.

## The roles of IFNs in ovarian cancer immunity

In early studies, researchers focused on the direct effects of IFNs on OC cells and ascites. With the increasing understanding of tumor immunity, people gradually began to realize the powerful regulatory role of IFNs on TME. And researches on finding the association between IFNs and tumor cells, immune cells and TME are gaining ground.

### The effects of IFNs on ovarian cancer cells

**Figure 2:**
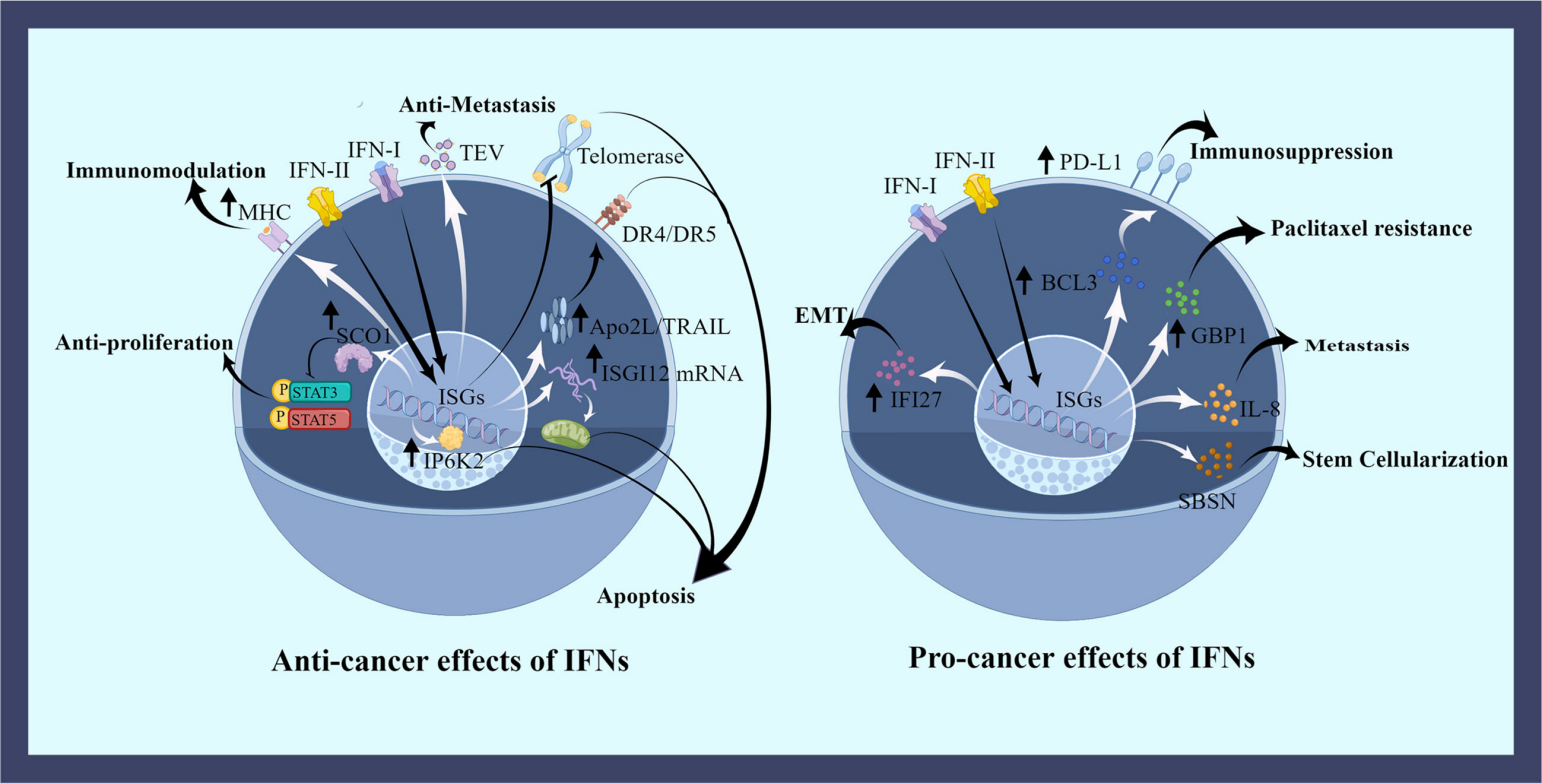
The effects of IFNs on ovarian cancer cells. IFNs have both favorable and unfavorable roles in OC cells. IFNs regulate OC cells by promoting the expression of some proteins or inhibiting some pathways. IFNs induce apoptotic effects in the nucleus, mitochondria, and cell membrane. Cell cycle is dysregulated by disrupting phosphorylation. The uptake of regulated tumor extracellular vesicles (TEVs) is inhibited by IFNs counteracting metastasis. And IFNs promote the expression of tumor antigens to aid immune killing. Many negative effects of the elevated protein expression caused by IFNs have been demonstrated, including OC cells metastasis, drug resistance, stemness, epithelial mesenchymal transition (EMT), and immunosuppression. This picture is drawn with the help of Figdraw.

To date, the role of IFNs on OC cells is highly controversial. On the one hand, IFNs can adversely affect the survival of OC cells, including their proliferation, metastasis, apoptosis and immune activation. On the other hand, IFNs favor the survival of OC cells, such as helping their growth, metastasis, and drug resistance (**Figure 2**).

In early studies, it was believed that IFNs had a positive effect on OC treatment. IFNs can induce apoptosis of OC cells directly through the death receptor-mediated pathway and mitochondrial pathway. And the expression of immune-related receptors on ovarian cancer cells is promoted, thus helping immune cells to detect OC cells. In experiments with inoculated NIH-OVCAR-3 cells to nude mouse, IFN-β was found to induce strong expression of Apo2L/TRAIL (Apo2L/tumor necrosis factor-associated apoptosis-inducing ligand) which can combine with death receptors DR4 (TRAIL-R1) and DR5 (TRAIL-R2) inducing apoptosis in OC cells (36, 37). IFN-β also increased human inositol hexakisphosphate kinase 2 (IP6K2) expression by post-transcriptionally regulation, which promoted apoptosis in the nucleus (38). High levels of ISG12A mRNA were found in stage III plasmacytoid OC. ISG12A mRNA impacted apoptosis through the mitochondrial intrinsic pathway, and the expression level of ISG12A in hepatocellular carcinoma was positively correlated with TRAIL-induced apoptosis (39–41). IFN-γ inhibited STAT3 and STAT5 protein phosphorylation pathways in a concentration-dependent manner by upregulating SOC1. In this way, OC cells proliferation, migration, cell cycle and invasion were impeded (42). IFN-α and IFN-γ blocked tumor cell growth and proliferation by reducing RNA synthesis, amino acid uptake and protein synthesis (43). Cell cycle arrest and cytotoxicity in OC cells were caused by HIFN-γ through activation of p53 and p21, leading to cell death (44). Cooperation of granzyme B, IFN-γ, and others from CD8^+^ T cells and NK cells directly killed OC cells (11, 45). In addition, IFN-β signaling oppressed telomerase activity and reverse transcriptase transcription in OC through the p21WAF1 pathway, which ultimately induced apoptosis in human OC cells (46). Studies have shown that pDCs induce immunosuppression and promote tumor growth in human OC and myeloma. Although the molecular mechanisms by which pDCs acquires these properties were unknown, it was interesting to note that human pDCs activated by CpG-containing DNA inhibited the growth of myeloma cells and induced apoptosis *via* producing IFN-α. Nevertheless, direct contact between myeloma cells and pDCs would degrade TLR9 of pDCs, which greatly reduced IFN-α expression, and promoted tumor progression (47). In Moreover, major histocompatibility complex (MHC) and tumor antigen expression on the surface of tumor cells were regulated by IFNs. However, this was related to the heterogeneity of cancer cells; some OC cells exposed to IFN-γ upregulated their cell surface MHC molecules and thus were killed by T cells, while some OC cells did not respond after stimulation by IFN-γ, and such hypo differentiated cells were killed by NK cells (48). IFNs affected not just the apoptosis and immunity of cancer cells, but also their metastasis. Regulated tumor extracellular vesicles (TEVs) secreted by tumors were able to degrade IFNAR1, thus inhibiting IFN-I, impairing ISGs expression and aiding in the formation of pre-metastatic ecological niches. And the uptake of TEVs were reduced by sustained IFN signaling, then metastatic process was compromised (49).

As people have studied more, however, it has been found that certain IFN signaling simultaneously contributes to the deterioration of OC cells. IFN-I responses were stimulated in tumor-prone cells of BRCA2 inactivated. Upregulation of ISGs resulted in cell cycle resumption. IFN responses help dying OC cells resume cell cycle and continue to proliferate (32). IFN-γ stimulated the release of full-length GBP1 in SKOV3 and OVCAR5 *via* a non-classical secretory pathway. GBP1 is a guanylate-binding protein with GTPase activity, which impedes cell production and angiogenesis. It inhibited tumor cell growth in breast cancer, and the expression of GBP1 in OC was associated with paclitaxel resistance, predicting a significantly shorter progression-free survival in OC (50–53). IFI27 was an overexpressing protein induced by IFN-α in OC tissues. It induced epithelial mesenchymal transition of OC cells and promoted migration and invasion of cancer cells (54). IFN-γ had the similar role, inducing IL-8 expression through JAK1/STAT1 signaling and p65 NFκB-mediated, thus helping OC cells to migrate (55, 56). In Addition to that, the synergistic effect of IFN-γ/JAK and ERK signaling pathways induced the expression of skin-specific protein suprabasin (SBSN) in OC cells, which reduced the adhesion of cancer cells and made them more resistant to apoptosis of lost nests, aiding OC cells metastasis and stem-cell-like property (57). PD-L1 expression can be induced either by OC cells or lymphocytes (58, 59). IFN-γ in OC cells amplified PD-L1 expression *via* JAK1/STAT1 and IRF1 signaling in a dose-dependent manner (60–62). Also, IFN-γ induced PD-L1 expression was also associated with Bcl3. IFN-γ was an agent that increased the expression of Bcl3 in OC cells, leading to increased transcriptional activity of PD-L1. PD-L1 expression was significantly reduced in OC cells stably transfected with Bcl3 shRNA (63, 64).

In summary, IFNs’ effects are variable. On the one hand, OC cells can be killed with IFNs through direct or indirect effects. And on the other hand, they support their survival. IFNs not only help the formation of cancer cells, but also their metastasis, immune escape and drug resistance.

### The effects of IFNs on immune cells in ovarian cancer microenvironment

It is well known that the presence of IFNs implies a pro-inflammatory tumor immune microenvironment. And a high level of IFNs generally facilitates better performance of immune cells. Abnormalities in tissues cause IFN responses that attracts immune cells and enhances their immune function to fight or clean up abnormal substances (65). IFNs initiate and promote immune responses that help the infiltration of immune cells, differentiation toward anti-tumor cells, activation of immune cells, and presentation of antigens, which are conducive to improving the warmth in the immune microenvironment of OC (**Figure 2**). BRCA1-deficient OC cells underwent chromatin reorganization and transcriptional reprogramming, a process that caused sensitization of the dsDNA-sensing pathway and excessive accumulation of cytoplasmic dsDNA, which then provoked an IFN response *via* the STING pathway, an inflammatory state that can aid in the recruitment of T cells and activation of DCs in tumor (30, 31, 66). CD4^+^ T (Th1) cells produced high levels of IFN-γ in response to antigenic stimulation, and then the secreted IFN-γ further enhanced Th1 cell development and stimulated macrophages to produce reactive oxygen nitrogen species and TNF-α to kill OC cells (15). Among patients with recurrent metastatic OC inoculated into mRNA-encoded folate-receptor-α transfected autologous DCs, the phenomenon of increased CD8^+^ and CD4^+^ T cells was observed due to the rise in the production of IFN-γ (67). EVs with abundant RNA induced IFN-I responses in human primitive monocyte and macrophage cell lines, which induced MHC-I expression for surveillance of the immune system and enriched immune promoting cells (29). Macrophages were induced to differentiate into M1-types with antitumor activity by lipopolysaccharide (LPS) *via* IFN-γ, and IFN signaling in ascites-associated macrophages was associated with good clinical outcomes in a subset of OC patients (68). It is believed that the cooperation between CCL5 and CXCL9 (IFNγ-inducible chemokine) could contribute to immune activation in OC. CCL5 is expressed by cancer cells and CXCL9 is produced by immune cells in cancer tissue. First, CCL5 produced by cancer cells attracted T cells into the tumor tissue, then T cells activated cancer antigens, then tumor antigens induced CXCL9 to secrete by immune cells dependent on the release of IFN-γ, and finally CXCL9 promotes further tumor infiltration by T cells. CCL5 and CXCL9 were linked by IFN-γ in a positive cycle that helped the establishment of hot tumors (69).

Correspondingly, abnormal factors that cause IFN signaling to be reduced or inhibited are detrimental to the infiltration and action of immune cells in cancerous tissue. PERK is an intermediate kinase of the unfolded protein response (UPR), which is activated at an elevated rate in malignant cells. It promoted immunosuppression of tumor Myeloid-derived suppressor cells (MDSCs) by stimulating the transcription factor NRF2. PERK also directly limited IFN-I responses by inducing phosphorylation-driven degradation of IFNAR1. The presence of PERK promoted immune suppression mediated by myeloid‐derived suppressor cells (70). Jiawen Zhang et al. found that the ubiquitinase USP35 was upregulated in OC tissues. Because USP35 is a negative regulator of STING-related IFN-I signaling, its high level was negatively correlated with CD8^+^ T cells, macrophage, neutrophil and DC infiltration in OC patients (34). In response, USP30 is a deubiquitinating enzyme on the outer mitochondrial membrane. The researchers found that sustained killing ability of TILs was reduced in USP30-deficient mouse, and its deletion led to mitochondrial abnormalities that affected the translation process of IFN-γ (71). More interestingly, when TILs express low levels of IFNs, this class of cells may have a suppressive effect on the TME. A study of OC patient specimens revealed that the percentage of γδ T cells was significantly higher in OC tissue than in marginal OC tissue and normal ovarian tissue. Also, there was a positive correlation between the higher number of γδ T cells in OC tissues and the advanced clinicopathological characteristics of OC patients. This is due to the comparatively low level of IFN-γ secreted by γδ T cells, a class of cells with weak cytotoxic effects and immunosuppressive activity in the OC microenvironment (13). Analysis of mRNA in peripheral blood lymphocytes stimulated by exosomes from malignant ascites revealed that malignant vesicles contributed to the formation of an immunosuppressive microenvironment within OC through IFN responses (72).

## The roles of interferons in OC treatment

**Figure 3:**
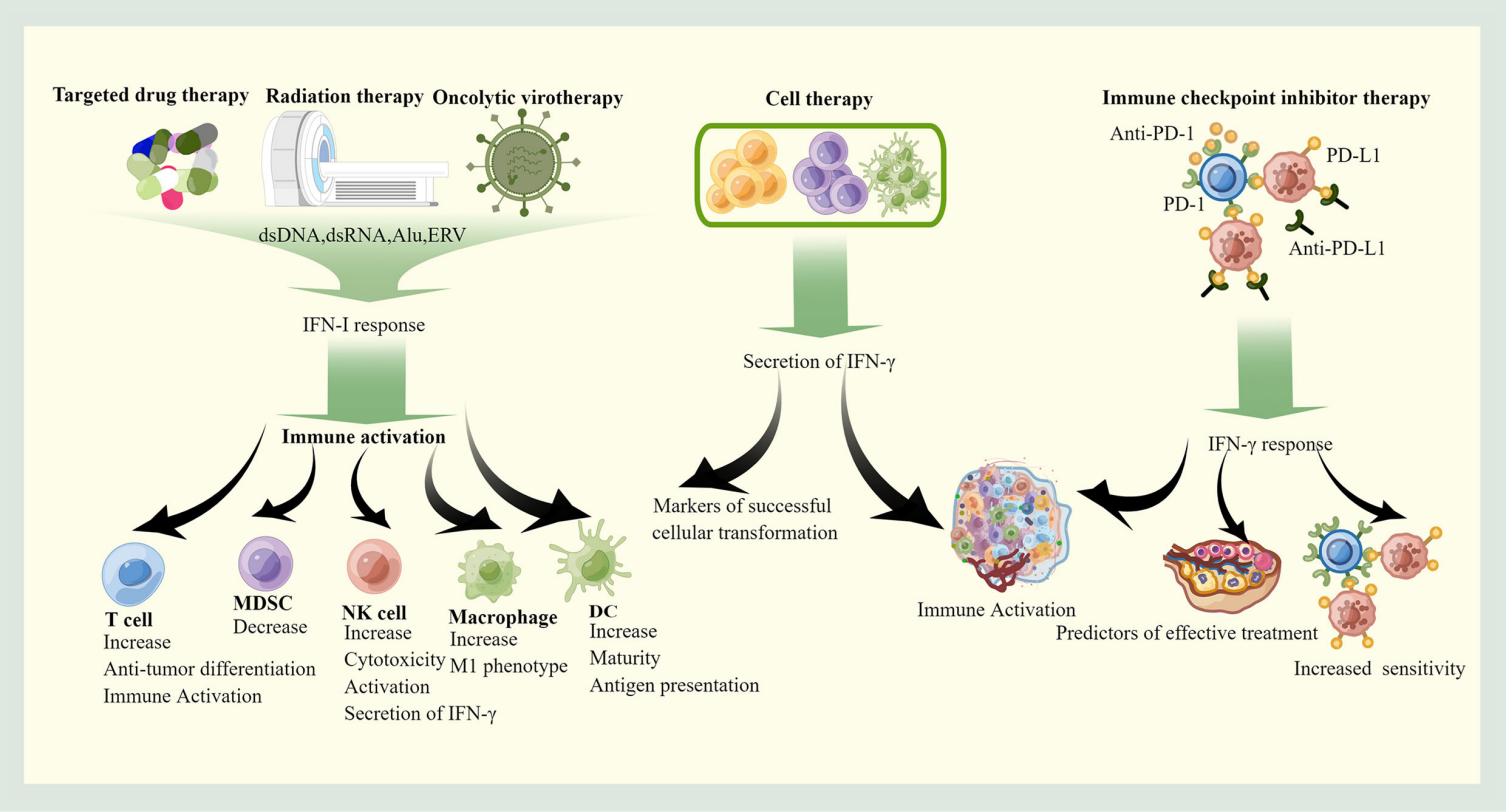
The roles of IFNs in therapy. Targeted therapy, radiotherapy and Oncolytic virotherapy stimulate the immune system of OC mainly through the IFN-I pathway, which promotes the recruitment and activation of pro-immune cells. Cell therapy can autonomously secrete IFN-γ to exert anti-cancer effects, and immune checkpoint inhibitor therapy requires indirect induction of IFN-γ production through feedback from immune checkpoints. The produced IFN-γ helps the formation of immune-friendly microenvironment, and high IFN-γ is associated with good prognosis. This picture is drawn with the help of Figdraw.

In most cases, the treatment of OC triggers IFN responses, which further enhance these therapeutic effects by modulating the immune system. IFN responses also may impede therapeutic effects at same time. With the exception of chemotherapy, which has little to do with immune activation of OC, other treatments act through immune activation and are associated with IFN response (**Figure 3**).

### Radiation therapy

Radiotherapy (RT) is a common and effective modality for treatment of OC to control cancer by inducing tumor cell death through DNA damage. Coincidentally, DNA damage activates cytoplasmic nucleic acid sensor pathways that induce inflammatory signals such as IFN responses, which reshape the immune environment of the TME (73). DNA damage from RT formed micronuclei and chromatin bridges in irradiated tumor cells and activated the cGAS/STING pathway, leading to IFN-I production. Thus, the antitumor effect of RT was abolished in IFN-I non-responsive hosts (74–76). This pathway was dependent on IFN-I signaling on DCs. IFN signal caused enhanced antigen uptake and presentation by DCs, which initiated naive T cells into an effector phenotype. STING-deficient mouse had much weaker CD8 T-cell response after radiation exposure than WT mouse, and IFN-β treatment was able to rescue the cross-initiation of cGAS or STING deficient DCs (77). In addition, radiotherapy-produced dsRNAs were involved in the IFN-I responses through the RLR family (78, 79). Notably, the RT-induced intrinsic IFN-I responses in cancer cells based on the dose and regimen. The DNA exonuclease Trex1 was able to attenuate the IFN-I responses by decreasing DNA that accumulated in the cytoplasmic matrix during RT. Therefore, it was important to adjust the RT dose that was just below the threshold for activation of Trex1 (80, 81). IFN-γ was also increased after RT. In the RT setting, IFN-γ have no significant direct function on tumor cells. However, comparison the TME under RT in IFN-γ deficient and wild mouse revealed that IFN-γ upregulated VCAM-1 expression of adhesion molecules on tumor vessels in the condition of local radiation, while increased IFN-γ induced chemokines CXCL9 and CXCL10, which contribute to the flow of activated T cells to irradiated tumors (82). In addition, RT enhanced the ability of T cells killing malignant cells in an IFN γ-dependent manner reducing the tumor burden (83). Combination therapy for high-dose RT ICB for OC have tried. Due to the diffuse spread of OC throughout the abdominal cavity, the abdominal viscera are exposed to the high toxicity risk of conventional RT. Follow-up studies found that RT with low-dose radiotherapy could also reprogram the TME (84, 85). Recent studies have shown that 1 Gy irradiation in whole abdominal RT in mouse with advanced OC was sufficient to induce important transcriptional changes *in vivo*, including IFN-α and IFN-γ responses, as well as cross-presenting DCs, which promoted T-cell infiltration (86). IFNs produced by the induction of RT create a positive TME for therapy.

### Chemotherapy

The current mainstream chemotherapy is based on paclitaxel and platinum for OC. However, some patients develop chemotherapy resistance in the late stage of treatment, which has been found to be tightly associated with the IFN responses. Part of the IFN responses resist chemotherapy resistance, while part of the responses contributes to the development of chemotherapy resistance. The release of glutathione and cysteine from tumor fibroblasts reduced the nuclear accumulation of platinum in OC cells, leading to resistance to platinum chemotherapy. CD8^+^ T cells secrete IFN-γ to control fibroblast glutathione and cysteine, thereby reducing fibroblast-mediated platinum resistance *in vivo* (87). Comparison of cisplatin-resistant OC cells with non-resistant cells indicated that ISGI15 (IFN-I induced) was reduced in resistant cells. Free ISIG15 increased the sensitivity of OC cells to cisplatin, and a decrease of ISGI15 was associated with poor prognosis (88). A study analyzing the metabolome and proteome in normal and resistant group OC cells revealed perturbations of IFN signaling in resistant group cancer cells. Tyrosine kinase 2 (TYK2), an enzyme that plays a key role in IFN-I signaling, was reduced in abundance in CAOV3 CBPR cells (89). This conclusion was similarly validated by clinical data showing that patients with high IFN-γ in the immune microenvironment had a better prognosis in platinum-treated patients (90). Furthermore, it has been shown that cancer cells pretreated by IFN-β were more likely to expose calreticulin and aided immunogenic cell death (ICD) when receiving platinum treatment through the IRF1 pathway (91). However, due to the numerous downstream regulatory pathways of IFNs, some pathways may contribute to drug resistance in OC cells. IFI16 belongs to the IFN-inducible PYHIN-200 gene family. Upregulation of IFI16 expression was observed in paclitaxel- resistant and adriamycin-resistant cell lines of OC. Though its real role had not been elucidated yet, it was speculated that it may involve in the regulation of drug-resistant gene expression (92). Meanwhile, extensive preclinical data suggested that many chemotherapies, including oxaliplatin, cisplatin, paclitaxel and 5-fluorouracil, promoted the upregulation of co-inhibitory ligands such as PD-L1. And this is usually a consequence of IFN-I or IFN-γ signaling, which together render drug ineffectiveness (93).

### Targeted drug therapy

Targeted drugs usually need to trigger IFN responses or depend on the IFN pathway to act. We will describe some drugs in targeted OC therapy that are associated with IFNs, such as poly (ADP) -ribose polymerase (PARP) inhibitors and epigenetic modulators.

Small molecule inhibitors of PARP (PARPi) have been approved for clinical use in the treatment of BRCA1 and BRCA2-deficient OC (94). The use of PARPi blocked the repair of single-stranded DNA damage in BRCA2-deficient cells, further promoting the exacerbation of high levels of DNA damage inherent in BRCA2-deficient cells. Antigen-presenting cells (APCs) sensed these accumulated dsDNA fragments, which drove the activation of IFN-I signaling, which would contribute to better anti-cancer immune action of PARPi (66, 95). A follow-up study found that PARPi was sensed by the cGAS-STING pathway *via* DNA and stimulated IFN-I production. And this process was independent of BRCA mutations, implying that ovarian cancer cells were triggered IFN-I responses by the use of PARPi (96). However, the secretion of total IFN-γ in immune cells treated with this drug was reduced. It was because of the significant increase in the activity of STAT3, which was a negative regulator of IFN-γ. Such negative regulation caused tumor resistance and immunosuppression (97). This also means that relying on direct regulation of IFNs by PARPi for may not be effective for anticancer effects due to the bidirectional regulation of PARPi for IFNs. Drugs affecting epigenetics such as AZA (dnmti 5-azacytidine), histone deacetylase inhibitor (HDACi), and etc. caused transcription of repeat elements, forming dsRNA and provoking IFN responses. In the OC model, a clear upregulation of inverted Alu repeats was found with AZA. Inverted Alu repeats can combine with MDA5 (dsRNA sensors), which provoked IFN signaling (98, 99). Additionally, AZA also acted as a DNA methyltransferase inhibitor, which can bind to both DNA and RNA, inhibiting the RNA methyltransferase. Thus, AZA demethylated RNA, and unmethylated RNA activated IFN-I. Through IFN-I signaling, AZA increased immune-promoting cells in the TME while reducing ascites and tumor burden, prolonging survival. If IFNAR1 was blocked, then IFN-I signaling would be restricted and the antitumor effect of AZA would also be limited (100). Stimulation of IFNs by AZA was enhanced in P53-mutant mouse (101). The combination of DNMTi and HDAC6i amplified IFN-I more than either one, reversing the immunosuppressive TME but also increasing PD-L1 expression on the cell surface. Overall, they perform only modest effects on survival (102). IFN-dependent antitumor immunity was stimulated by an increase in endogenous retroviral elements (ERV) induced by inhibition of histone lysine specific demethylase 1 (LSD1). SP-2577 is an LSD1 inhibitor. SP-2577 promoted ERV expression in ovarian cancer cells and activated the dsRNA-induced IFN pathway, which facilitated cytokine expression and infiltration of immune cells in ovarian hypercalcemic organoids (103). CX-5461, an RNA polymerase I inhibitor, led to the accumulation of cytoplasmic dsDNA activating the cGAS-STING-TBK1-IRF3 innate immune pathway, which induced IFN-I and promoted the secretion of IL-6 and CXCL10, contributing to T cell infiltration (104). These drugs not only act directly on cancer cells, but also stimulate the IFN through their subsequent chain reaction, affecting the immune microenvironment and causing a continuous anti-cancer effect.

### Immunotherapy

Research on immunotherapy is promising in the treatment of OC, especially for immune checkpoint blockade therapy. Immune checkpoints and IFNs are closely linked due to the fact that changes in immune checkpoints trigger IFN responses and IFN responses also modulate immune checkpoints. At the same time, IFNs serve as predictors of immune checkpoint blockade treatment status. Clinical studies have found that the release of IFN-γ was significantly increased when TILs isolated from OC patients were co-incubated with PD-1 antibodies, reversing the immunosuppressed OC microenvironment to some extent (22, 105). *In vivo* imaging to track intra-tumor factor changes after a PD-1 treatment, and single-cell sequencing validation suggest that the process of successful anti-PD-1 cancer immunotherapy performs as follows. T cells secreted IFN-γ to activate DCs to release IL-12, and then IL-12 stimulated T cells to continue to secrete IFN-γ and activated other immune cells, and then a positive circulating immune response was established within the tumor, ultimately leading to tumor killing (106). At the same time, IFN-I helped carboplatin-treated HGSC sensitize to immune checkpoint blockade therapy *via* STING pathway, and studies have also found that tumor cell resistance to immune checkpoint blocking drugs was associated with reduced sensitivity to IFN-γ signaling or loss of IFN signaling (107, 108). IFNs do not merely have an important effect on PD-1, but also have a close relationship with PD-L1. When PD- L1 knockout (KO) and control OC cells were inoculated intraperitoneally into syngeneic mouse, the PD- L1- KO group showed a significant increase of CD4^+^ T cells, CD8^+^ T cells, NK cells and CD11c^+^ (M1-like) macrophages compared to the control group, and Th1-type cytokines such as IFN-γ were also significantly increased (109). PD-L1 blockade was also capable of increase IFN-γ secretion, and the phenomenon was more pronounced after combination with the pyruvate dehydrogenase kinase inhibitor dichloroacetate (DCA) (23, 110–112). More importantly, it has been shown that resistance to immune checkpoints and loss of IFN-γ signaling were related. Interruption of IFN-γ signaling prevented the induction of PD-L1 expression, rendering PD1-PD-L1 blockade ineffective (113, 114). A statistical review of clinical study data revealed that basal serum IFN-γ levels were associated with disease control rates and overall survival in cancer patients. Patients with high IFN-γ showed better performance in immune checkpoint inhibitor therapy (115). The analysis also suggested that IFN-γ, or IFN-α, IFN-γ, IL-2 combined with TNF-α secretion could predict the efficacy of PD-1 inhibitors in cancer patients (116, 117).

In addition to the immune checkpoints mentioned above, there are some immunomodulatory drugs which also exert anti-cancer effects by modulating the TME through IFN signaling. IL-15 super agonist (N-803) increases NK cell proliferation and IFN-γ production, overcoming the defective NK cell function caused by the immunosuppression of OC. Co-culture with N-803, NK cells and OC-like organs revealed that N-803 increased IFN-γ secretion from NK cells and further increased IFN-γ induced CXCL10 secretion enhancing NK cell cytotoxicity against OC (110, 118). CDK4/6 expression was higher in OC tissues than in normal ovarian tissues. High CDK4/6 expression resulted in an immunosuppressed state in OC and was associated with poor prognosis for OC patients. The CDK4/6 inhibitor palbociclib activated immunity in OC by increasing the secretion of IFN-γ and the expression of ISGs, which upregulated the expression of antigen-presenting molecules (119).

### Cell therapy

Cell therapies such as CAR-T therapy, NK cell treatment, and DC vaccines have been extensively studied in OC clinical trials. CAR-T cells enhance cytotoxic effects on malignant cells by specifically recognizing surface antigens on tumors and secreting cytokines like IFN-γ. And then, IFN-γ can inhibit tumor progression by promoting the secretion of downstream cytokines, infiltration of T cell and avascular necrosis (120, 121). 5T4 is a tumor-associated antigen that is actively expressed on the cell surface of most solid tumors, including OC. Effective transduction of patient T cells with anti-5T4 CAR and antigen-specific secretion of IFN-γ was produced by the co-culture of CAR-T cells and matched autologous tumor catabolites. And IFN-γ production correlated with tumor cell surface 5T4 expression levels (122, 123). In addition, CAR-T therapy modulated the differentiation of T cells through IFN-γ affecting tumor immunity (124). One study constructed CAR-T cells with a lentiviral vector, which released a large number of cytokines, such as IL-2, IFN-γ and TNF-α, to activate T cells and NK cells to promote massive release of factors. The CAR-T demonstrated a strong killing ability against OVCAR-3 cells *in vitro* (125). However, results in hematological malignancies showed that IFN-γ was not required for the efficacy of CAR-T, and that IFN-γ inhibition reduced the secretion of other toxic factors and improved the efficacy and clinical durability of CAR-T (126). IFN also can be used alone or in combination with other compounds to mature DCs in ex vivo production (127). Because of the secretion of IFN-γ of DCs and NK cells is one of the indicators of successful construction. Injecting these cells into the body induced therapeutic immunomodulatory effects by Continuously releasing IFN-γ (67, 128–130). In conclusion, IFNs play an important role in cell therapy. The function of IFNs is not only as participant in cell construction, but also as indispensable worker in treatment.

### Oncolytic virotherapy

Oncolytic viruses (OV) can exert a therapeutic effect by direct lysis of tumor cells, or transporting therapeutic genes. OC cell lines are capable of making an IFN-I response to induce an antiviral state upon viral infection, which is in the contrast to other cancer cell lines. In the treatment of OV, there is a mutual resistance in treatment of OV between the virus and the IFNs. On the one hand, IFNs block and clear the OV in the organism so that the OV is ineffective against the tumor. On the other hand, the OV stimulates the IFN response, which activates immunity to operate against the tumor. The IFN response inhibited the vesicular stomatitis virus-glycoprotein (VSV-GP), shifting cancer cells to an antiviral state and making them resistant to VSV-mediated tumor lysis. This inhibition can be reversed as IFN signaling was regulated with the Jak1/2 inhibitor ruxolitinib (131). Despite the fact that IFN responses impede the action of OV, OV-induced IFN secretion may recruit more immune cells. Lysing adenovirus delivering TNF-α and IL-2 was found to increase IFN-γ secretion in isolated OC tissues, accompanied by T cell activation, promoting infiltration of tumor lymphocytes in the OC microenvironment (132). Defective IFN signaling may be more conducive to OV sensitivity. Cells with the disruption of innate antiviral defenses associated with IFN were more vulnerable to viruses. In detail, cell sensitivity to viral infection was associated with expression level of IFNAR, genes induced by IFNs, pattern recognition receptors and JAK/STAT pathway (133). And this was proved in many malignant cells (134–136). More interestingly, IFN-I secreted by pDCs enhanced the lytic activity of non-replicating HSV-1d106S (137).

## Application of IFN response

Researches on the utilization of IFNs for the treatment of OC have been under way for decades. Indeed, effective treatment with IFNs in OC has been tried, comprising the use of IFNs for the direct treatment of OC, the combination of IFN therapy with other therapies, and treatment with the help of recovery the IFN response in OC. Some clinical trials related to IFN response in OC treatment in recent years are shown in **Table 1**.

**Table 1:** The clinical trials based on IFN response in OC.

Type	Combination Therapy	Disease	Phase	Status	Reference	Remark
IFN-α	Carboplatin+Paclitaxel+TILs		I/II	Recruiting	NCT04072263	IFN α-2b
	Cisplatin+Celecoxib+ DC Vaccine		I/II	Suspended	NCT02432378^[147]^	A cocktail of rintatolimod and IFN-α
	Denileukin Diftitox	EOC	II	Terminated	NCT01773889	PEG-IFN α-2b
		EOC	I/II	Terminated	NCT00085384	PEG-IFN α-2b
	Gemcitabine+P53 SLP Vaccin	Recurrent OC	I/II	Completed	NCT01639885	PEG-IFN α-2b
	Radiolabeled Monoclonal Antibody+Paclitaxel		I	Completed	NCT00002734	Recombinant IFN-α
	Carboplatin +Doxorubicin+ Tocilizumab	Recurrent OC	I/II	Completed	NCT01637532	PEG-IFN α-2b
	IL-2+Sargramostim		II	Completed	NCT00003408	Recombinant IFN-α
IFN-β	Recombinant Adenovirus-hIFN-β	Cancer	I	Completed	NCT00066404	
IFN-γ	Carboplatin And Paclitaxel		III	Terminated	NCT00047632	IFN γ-1b
	Tumor Vaccine	Recurrent and Epithelial OC	I	Completed	NCT00004032	
	GM-CSF+Carboplatin		II	Completed	NCT00501644	
IFN-α IFN-γ	Autologous Monocytes		I	Terminated	NCT02948426^[141]^	PEG-IFN α-2b
Activator of STING	Pembrolizumab	Advanced Solid Tumor	I	Recruiting	NCT04609579	SNX281
IFN	Recombinant L-IFN Adenovirus Injection		I	Recruiting	NCT05180851	

IFN, interferon; OC, ovarian cancer; EOC, Epithelial ovarian cancer; TILs, Tumor infiltrating lymphocytes. Blanks in the disease column refer to ovarian cancer. Blanks in the remark column refer to subtypes of interferon that are not specified.

Due to the powerful effects of IFNs on tumor cells and immune microenvironment, many works have been attempted to apply IFNs directly to OC therapy. But the metabolic characteristics of IFNs and the prevalent expression of IFN receptors have limited their application. Attempting local administration and modification of IFNs to improve their bioavailability and targeting is a research trend. For example, coupling IFNs with polyethylene glycol, hyaluronic acid, or aluminum salts not only prolonged the duration of action but also improved drug targeting, allowing them to be trapped in the peritoneal cavity or in the tumor (138–141). In addition, bone marrow mononuclear cells (iPS-ML) have been genetically modified to produce IFN-β. This class of cells reduced cancer cells in an iPS-ML/IFN-β dose-dependent way when co-cultured with OC cells. When injected into OC mouse with ascites, iPS-ML/IFN-β infiltration into the cancerous tissues was observed and cancer-associated ascites was dramatically reduced (142).

In addition to structural modification and exogenous introduction of IFNs to treat ovarian cancer, combining IFNs with other modalities to adjuvant therapy, especially with immunotherapy to improve the suppressive immune microenvironment of OC is a prospective therapeutic tool for establishing hot tumors. The TLR4 agonist MPLA stimulated IFN-I signaling in combination with IFN-γ. Activated macrophages and cytotoxic T cells by IFN-I reversed immunosuppression, prolonged the median survival of tumor-bearing mouse and inhibited metastatic progression of OC (143). The binding of IL-4-PE, IFN and IFN caused increased cell death for both OC cells *in vitro* and *in vivo*, increasing tumor-bearing mouse survival. Mechanistically, the synergistic antitumor effect was dependent on IFN signaling, and key proteins activated by both IFNs and IL-4-PE had a critical role in the apoptotic pathway (144). Monocytes have been shown to be cytotoxic to tumor cells in the absence of pro-inflammatory cytokines. In mouse models, stimulation of monocytes with IFN-α and IFN-γ resulted in a significant reduction in tumor volume and an increase in overall survival, which was achieved by modulating intra-tumor immunity (145). During clinical trials, autologous monocytes were stimulated with IFNs *in vitro* and then injected into the peritoneal cavity of patients with advanced chemotherapy-resistant OC. The results showed that IFN α-2a or IFN γ-1b had potent antitumor effects *in vitro* and *in vivo*, and their effects were multiplied with the addition of monocytes (146). Combining adriamycin and IFN-β, IFN-β can promote DOX-mediated cell death (147). A recent study used IFN-γ to transport DOX in the form of nanoparticles. Such nanoparticles greatly increased apoptosis at the cellular level (148).

The blockage of some IFN signaling promotes the development of cold tumors in OC, hence enhancing IFN signaling would facilitate the development of hot tumors as well. PARP7 is a member of the mono-PARP class of enzymes and blocks IFN response by inhibiting nucleic acid sensing. RBN-2397 is a potent and selective inhibitor of PARP7. In preclinical models, RBN-2397 restored IFN-I signaling in tumors, inhibited cancer cell proliferation and induced adaptive immunity, leading to tumor regression (149). In later experiments in patients with advanced solid tumors, an increase in the expression of ISGs and an enrichment of the immune response gene, accompanied by an increment in CD8^+^ T cells, was observed on tumor biopsies after the use of RBN-2397 (150). Moreover, it was found that Gal-3 secreted by tumor cells or stromal cells bound to N-glycans, forming a glycoprotein/Gal-3 lattice that accumulates in the TME and intercepts glycosylated soluble factors, particularly IFN-γ. As IFN-γ diffusion was restricted in OC, CXCL9/10 concentration decreased, which facilitated “cold” tumor phenotypes by limiting T-cell infiltration. In DCs-rich plasma OC models, the combination of G3-C12@PLGA (Gal-3 antagonist) and anti-PD- 1 peptide was effective. G3-C12@PLGA not only maintained CXCL9/10 concentrations in tumor tissues for a long time by releasing IFN-γ and continuously recruiting CD8^+^ T cells into tumors, but also helped anti-PD-1 peptide to function better. The combination of the two significantly inhibited the increase of ascites, reduced the metastasis of peritoneal tumors, prolonged the survival of model mice, and offered the possibility of a cure for OC (151).

## Conclusion and outlook

Currently, we are still in an accumulation phase where people are studying the detailed mechanisms of IFNs in OC, both in terms of its action on OC and its regulation by OC. Although the IFN responses have a bidirectional role for OC development, high levels of IFNs are more prognostic, which implies that there is a game in the TME in which the protein produced by the IFNs that favors therapy ultimately dominates. The role of IFNs is a hinderer to an extent in the OC development.

Based on the summary of the literature, there is still confidence in the use of IFNs for the treatment of OC. OC is a cold tumor, and IFNs can change it to a hot tumor, allowing a more active immune environment that is conducive to immunotherapy. And this also provides ideas for other cold tumors. The combination therapy with IFN and cell therapy is a promising research direction. The use of IFNs in OC, however, is worthy of serious deliberating, taking into consideration of the time, dose, and site of use, which are closely related to the therapeutic effect. In addition, due to the numerous response sites of IFNs, the side effects of IFNs should be considered. Selective activation of downstream targets or inhibition of some negative loci may be feasible approaches. At the same time, IFNs serve as presites for polygenic regulation, and we should also consider gene mutations to prevent ineffectiveness or false activation. In conclusion, the roles of IFNs in OC are complex and meaningful. And the combination of IFNs with other therapies is still a field deserving exploring.

## Author contributions

TL wrote the manuscript. YL, XW, XY, YF and YZ performed the work of review. HG and ZH designed the work of review. All authors contributed to the article and approved the submitted version.

## References

[1] Menon U Gentry-Maharaj A Burnell M Singh N Ryan A Karpinskyj C . Ovarian cancer population screening and mortality after long-term follow-up in the UK collaborative trial of ovarian cancer screening (UKCTOCS): a randomised controlled trial. Lancet (2021) 397:2182–93. doi: 10.1016/S0140-6736(21)00731-5 PMC8192829 33991479

[2] Zhang S Cheng C Lin Z Xiao L Su X Zheng L . The global burden and associated factors of ovarian cancer in 1990–2019: findings from the global burden of disease study 2019. BMC Public Health (2022) 22:1455. doi: 10.1186/s12889-022-13861-y 35907822 PMC9339194

[3] Mohr P Hauschild A Trefzer U Enk A Tilgen W Loquai C . Intermittent high-dose intravenous interferon Alfa-2b for adjuvant treatment of stage III melanoma: Final analysis of a randomized phase III dermatologic cooperative oncology group trial. J Clin Oncol (2015) 33:4077–84. doi: 10.1200/JCO.2014.59.6932 26503196

[4] Parker BS Rautela J Hertzog PJ . Antitumour actions of interferons: implications for cancer therapy. Nat Rev Cancer (2016) 16:131–44. doi: 10.1038/nrc.2016.14 26911188

[5] Hawkins RE Gore M Shparyk Y Bondar V Gladkov O Ganev T . A randomized phase II/III study of naptumomab estafenatox + IFNα versus IFNα in renal cell carcinoma: Final analysis with baseline biomarker subgroup and trend analysis. Clin Cancer Res (2016) 22:3172–81. doi: 10.1158/1078-0432.CCR-15-0580 26851187

[6] Galvani DW Cawley JC . The current status of interferonα in haemic malignancy. Blood Rev (1990) 4:175–80. doi: 10.1016/0268-960X(90)90045-T 2245253

[7] Eigentler TK Gutzmer R Hauschild A Heinzerling L Schadendorf D Nashan D . Adjuvant treatment with pegylated interferon α-2a versus low-dose interferon α-2a in patients with high-risk melanoma: a randomized phase III DeCOG trial. Ann Oncol (2016) 27:1625–32. doi: 10.1093/annonc/mdw225 27287206

[8] Beatty GL Paterson Y . Regulation of tumor growth by IFN-γ in cancer immunotherapy. IR (2001) 24:201–10. doi: 10.1385/IR:24:2:201 11594457

[9] Yu R Zhu B Chen D . Type I interferon-mediated tumor immunity and its role in immunotherapy. Cell Mol Life Sci (2022) 79:191. doi: 10.1007/s00018-022-04219-z 35292881 PMC8924142

[10] Foord E Klynning C Schoutrop E Förster JM Krieg J Mörtberg A . Profound functional suppression of tumor-infiltrating T-cells in ovarian cancer patients can be reversed using PD-1-Blocking antibodies or DARPin® proteins. J Immunol Res (2020) 2020:1–12. doi: 10.1155/2020/7375947 PMC7424497 32832572

[11] Scheper W Kelderman S Fanchi LF Linnemann C Bendle G de Rooij MAJ . Low and variable tumor reactivity of the intratumoral TCR repertoire in human cancers. Nat Med (2019) 25:89–94. doi: 10.1038/s41591-018-0266-5 30510250

[12] Gocher AM Workman CJ Vignali DAA . Interferon-γ: teammate or opponent in the tumour microenvironment? Nat Rev Immunol (2022) 22:158–72. doi: 10.1038/s41577-021-00566-3 PMC8688586 34155388

[13] Chen X Shang W Xu R Wu M Zhang X Huang P . Distribution and functions of γδ T cells infiltrated in the ovarian cancer microenvironment. J Transl Med (2019) 17:144. doi: 10.1186/s12967-019-1897-0 31064389 PMC6505080

[14] Sawada M Goto K Morimoto-Okazawa A Haruna M Yamamoto K Yamamoto Y . PD-1^+^ Tim3^+^ tumor-infiltrating CD8 T cells sustain the potential for IFN-γ production, but lose cytotoxic activity in ovarian cancer. Int Immunol (2020) 32:397–405. doi: 10.1093/intimm/dxaa010 32009163

[15] Kennedy R Celis E . Multiple roles for CD41 T cells in anti-tumor immune responses. Immunol Rev (2008) 222:129–44. doi: 10.1111/j.1600-065X.2008.00616.x 18363998

[16] Song M Sandoval TA Chae C-S Chopra S Tan C Rutkowski MR . IRE1α–XBP1 controls T cell function in ovarian cancer by regulating mitochondrial activity. Nature (2018) 562:423–8. doi: 10.1038/s41586-018-0597-x PMC6237282 30305738

[17] Wei X Jin Y Tian Y Zhang H Wu J Lu W . Regulatory b cells contribute to the impaired antitumor immunity in ovarian cancer patients. Tumor Biol (2016) 37:6581–8. doi: 10.1007/s13277-015-4538-0 26638169

[18] Gao Y Lu J Zeng C Yang J Huang B Zhang N . IL-10 suppresses IFN-γ-mediated signaling in lung adenocarcinoma. Clin Exp Med (2020) 20:449–59. doi: 10.1007/s10238-020-00626-3 32306136

[19] Betella I Turbitt WJ Szul T Wu B Martinez A Katre A . Wnt signaling modulator DKK1 as an immunotherapeutic target in ovarian cancer. Gynecologic Oncol (2020) 157:765–74. doi: 10.1016/j.ygyno.2020.03.010 32192732

[20] Pesce S Tabellini G Cantoni C Patrizi O Coltrini D Rampinelli F . B7-H6-mediated downregulation of NKp30 in NK cells contributes to ovarian carcinoma immune escape. OncoImmunology (2015) 4:e1001224. doi: 10.1080/2162402X.2014.1001224 26137398 PMC4485754

[21] Maas RJ Hoogstad-van Evert JS van der Meer JM Mekers V Rezaeifard S Korman AJ . TIGIT blockade enhances functionality of peritoneal NK cells with altered expression of DNAM-1/TIGIT/CD96 checkpoint molecules in ovarian cancer. OncoImmunology (2020) 9:1843247. doi: 10.1080/2162402X.2020.1843247 33224630 PMC7657585

[22] Rådestad E Klynning C Stikvoort A Mogensen O Nava S Magalhaes I . Immune profiling and identification of prognostic immune-related risk factors in human ovarian cance. OncoImmunology (2019) 8:e1535730. doi: 10.1080/2162402X.2018.1535730 30713791 PMC6343785

[23] Wang J-J Siu MK Jiang Y-X Leung TH Chan DW Cheng R-R . Aberrant upregulation of PDK1 in ovarian cancer cells impairs CD8^+^ T cell function and survival through elevation of PD-L1. OncoImmunology (2019) 8:1659092. doi: 10.1080/2162402X.2019.1659092 PMC6791447 31646108

[24] Shenoy GN Loyall J Maguire O Iyer V Kelleher RJ Minderman H . Exosomes associated with human ovarian tumors harbor a reversible checkpoint of T cell responses. Cancer Immunol Res (2018) 6:236–47. doi: 10.1158/2326-6066.CIR-17-0113 PMC5845437 29301753

[25] Stary G Bangert C Tauber M Strohal R Kopp T Stingl G . Tumoricidal activity of TLR7/8-activated inflammatory dendritic cells. J Exp Med (2007) 204:1441–51. doi: 10.1084/jem.20070021 PMC2118597 17535975

[26] Gungor B Yagci FC Tincer G Bayyurt B Alpdundar E Yildiz S . CpG ODN nanorings induce IFNα from plasmacytoid dendritic cells and demonstrate potent vaccine adjuvant activity. Sci Trans Med (2014) 6:235ra61–235ra61. doi: 10.1126/scitranslmed.3007909 24807558

[27] Labidi-Galy SI Sisirak V Meeus P Gobert M Treilleux I Bajard A . Quantitative and functional alterations of plasmacytoid dendritic cells contribute to immune tolerance in ovarian cancer. Cancer Res (2011) 71:5423–34. doi: 10.1158/0008-5472.CAN-11-0367 21697280

[28] Chae C-S Sandoval TA Hwang S-M Park ES Giovanelli P Awasthi D . Tumor-derived lysophosphatidic acid blunts protective type I interferon responses in ovarian cancer. Cancer Discovery (2022) 12:1904–21. doi: 10.1158/2159-8290.CD-21-1181 PMC9357054 35552618

[29] Porter RL Sun S Flores MN Berzolla E You E Phillips IE . Satellite repeat RNA expression in epithelial ovarian cancer associates with a tumor-immunosuppressive phenotype. J Clin Invest (2022) 132:e155931. doi: 10.1172/JCI155931 35708912 PMC9374379

[30] Cardenas H Jiang G Thomes Pepin J Parker JB Condello S Nephew KP . Interferon-γ signaling is associated with BRCA1 loss-of-function mutations in high grade serous ovarian cancer. NPJ Precis Oncol (2019) 3:32. doi: 10.1038/s41698-019-0103-4 31840082 PMC6897992

[31] Bruand M Barras D Mina M Ghisoni E Morotti M Lanitis E . Cell-autonomous inflammation of BRCA1-deficient ovarian cancers drives both tumor-intrinsic immunoreactivity and immune resistance *via* STING. Cell Rep (2021) 36:109412. doi: 10.1016/j.celrep.2021.109412 34289354 PMC8371260

[32] Reisländer T Lombardi EP Groelly FJ Miar A Porru M Di Vito S . BRCA2 abrogation triggers innate immune responses potentiated by treatment with PARP inhibitors. Nat Commun (2019) 10:3143. doi: 10.1038/s41467-019-11048-5 31316060 PMC6637138

[33] Cetintas VB Batada NN . Is there a causal link between PTEN deficient tumors and immunosuppressive tumor microenvironment? J Transl Med (2020) 18:45. doi: 10.1186/s12967-020-02219-w 32000794 PMC6993356

[34] Zhang J Chen Y Chen X Zhang W Zhao L Weng L . Deubiquitinase USP35 restrains STING-mediated interferon signaling in ovarian cancer. Cell Death Differ (2021) 28:139–55. doi: 10.1038/s41418-020-0588-y PMC7853139 32678307

[35] Schmitt M Gallistl J Schüler-Toprak S Fritsch J Buechler C Ortmann O . Anti-tumoral effect of chemerin on ovarian cancer cell lines mediated by activation of interferon alpha response. Cancers (2022) 14:4108. doi: 10.3390/cancers14174108 36077645 PMC9454566

[36] Morrison BH Tang Z Jacobs BS Bauer JA Lindner DJ . Apo2L/TRAIL induction and nuclear translocation of inositol hexakisphosphate kinase 2 during IFN-β-induced apoptosis in ovarian carcinoma. Biochem J (2005) 385:595–603. doi: 10.1042/BJ20040971 15634191 PMC1134734

[37] Green DS Ning F Duemler A Myers TG Trewitt K Ekwede I . Intraperitoneal monocytes and interferons as a novel cellular immunotherapy for ovarian cancer: mechanistic characterization and results of a phase I clinical trial. Clin Cancer Res (2022), CCR–22-1893:28. doi: 10.1158/1078-0432.CCR-22-1893

[38] Morrison BH Bauer JA Kalvakolanu DV Lindner DJ . Inositol hexakisphosphate kinase 2 mediates growth suppressive and apoptotic effects of interferon-β in ovarian carcinoma cells. J Biol Chem (2001) 276:24965–70. doi: 10.1074/jbc.M101161200 PMC2025680 11337497

[39] Gytz H Hansen MF Skovbjerg S Kristensen ACM Hørlyck S Jensen MB . Apoptotic properties of the type 1 interferon induced family of human mitochondrial membrane ISG12 proteins. Biol Cell (2017) 109:94–112. doi: 10.1111/boc.201600034 27673746

[40] Kim Y-S Hwan Do J Bae S Bae D-H Shick Ahn W . Identification of differentially expressed genes using an annealing control primer system in stage III serous ovarian carcinoma. BMC Cancer (2010) 10:576. doi: 10.1186/1471-2407-10-576 20969748 PMC2974737

[41] Liu N Long Y Liu B Yang D Li C Chen T . ISG12a mediates cell response to Newcastle disease viral infection. Virology (2014) 462–463:283–94. doi: 10.1016/j.virol.2014.06.014 24999841

[42] Gao AH Hu YR Zhu WP . IFN-γ inhibits ovarian cancer progression *via* SOCS1/JAK/STAT signaling pathway. Clin Transl Oncol (2022) 24:57–65. doi: 10.1007/s12094-021-02668-9 34275119

[43] Bromberg JF Horvath CM Wen Z Schreiber RD Darnell JE . Transcriptionally active Stat1 is required for the antiproliferative effects of both interferon alpha and interferon gamma. Proc Natl Acad Sci USA (1996) 93:7673–8. doi: 10.1073/pnas.93.15.7673 PMC38805 8755534

[44] Razaghi A Villacrés C Jung V Mashkour N Butler M Owens L . Improved therapeutic efficacy of mammalian expressed-recombinant interferon gamma against ovarian cancer cells. Exp Cell Res (2017) 359:20–9. doi: 10.1016/j.yexcr.2017.08.014 28803068

[45] Hoogstad-van Evert JS Maas RJ van der Meer J Cany J van der Steen S Jansen JH . Peritoneal NK cells are responsive to IL-15 and percentages are correlated with outcome in advanced ovarian cancer patients. Oncotarget (2018) 9:34810–20. doi: 10.18632/oncotarget.26199 PMC6205176 30410679

[46] Lee J-H Lee S-Y Lee J-H Lee S-H . p21WAF1 is involved in interferon-β- induced attenuation of telomerase activity and human telomerase reverse transcriptase (hTERT) expression in ovarian cancer. Mol Cells (2010) 30:327–33. doi: 10.1007/s10059-010-0131-y 20814747

[47] Bi E Li R Bover LC Li H Su P Ma X . E-cadherin expression on multiple myeloma cells activates tumor-promoting properties in plasmacytoid DCs. J Clin Invest (2018) 128:4821–31. doi: 10.1172/JCI121421 PMC6205396 30277474

[48] Chovatiya N Kaur K Huerta-Yepez S Chen P-C Neal A DiBernardo G . Inability of ovarian cancers to upregulate their MHC-class I surface expression marks their aggressiveness and increased susceptibility to NK cell-mediated cytotoxicity. Cancer Immunol Immunother (2022), 71:2929–2941. doi: 10.1007/s00262-022-03192-7 PMC10991924 35507102

[49] Kenific CM Wang G Lyden D . Tumor extracellular vesicles impede interferon alert responses. Cancer Cell (2019) 35:3–5. doi: 10.1016/j.ccell.2018.12.006 30645974

[50] Guenzi E . The guanylate binding protein-1 GTPase controls the invasive and angiogenic capability of endothelial cells through inhibition of MMP-1 expression. EMBO J (2003) 22:3772–82. doi: 10.1093/emboj/cdg382 PMC169055 12881412

[51] Lipnik K Naschberger E Gonin-Laurent N Kodajova P Petznek H Rungaldier S . Interferon γ–induced human guanylate binding protein 1 inhibits mammary tumor growth in mice. Mol Med (2010) 16:177–87. doi: 10.2119/molmed.2009.00172 PMC2864808 20454519

[52] Wadi S Tipton AR Trendel JA Khuder SA Vestal DJ . hGBP-1 expression predicts shorter progression-free survival in ovarian cancers, while contributing to paclitaxel resistance. J Cancer Ther (2016) 7:994–1007. doi: 10.4236/jct.2016.713097 28090373 PMC5226657

[53] Carbotti G Petretto A Naschberger E Stürzl M Martini S Mingari MC . Cytokine-induced guanylate binding protein 1 (GBP1) release from human ovarian cancer cells. Cancers (Basel) (2020) 12:488. doi: 10.3390/cancers12020488 32093058 PMC7072386

[54] Li S Xie Y Zhang W Gao J Wang M Zheng G . Interferon alpha-inducible protein 27 promotes epithelial–mesenchymal transition and induces ovarian tumorigenicity and stemness. J Surg Res (2015) 193:255–64. doi: 10.1016/j.jss.2014.06.055 25103640

[55] Vancurova I Zhu Y Springer US . Immune mediators in cancer: Methods and protocols. New York, NY (2020). doi: 10.1007/978-1-0716-0247-8

[56] Padmanabhan S Gaire B Zou Y Uddin MM DeLeon D Vancurova I . IFNγ induces JAK1/STAT1/p65 NFκB-dependent interleukin-8 expression in ovarian cancer cells, resulting in their increased migration. Int J Biochem Cell Biol (2021) 141:106093. doi: 10.1016/j.biocel.2021.106093 34626802 PMC8639749

[57] Hubackova S Pribyl M Kyjacova L Moudra A Dzijak R Salovska B . Interferon-regulated suprabasin is essential for stress-induced stem-like cell conversion and therapy resistance of human malignancies. Mol Oncol (2019) 13:1467–89. doi: 10.1002/1878-0261.12480 PMC6599850 30919591

[58] Abiko K Matsumura N Hamanishi J Horikawa N Murakami R Yamaguchi K . IFN-γ from lymphocytes induces PD-L1 expression and promotes progression of ovarian cancer. Br J Cancer (2015) 112:1501–9. doi: 10.1038/bjc.2015.101 PMC4453666 25867264

[59] Abiko K Hamanishi J Matsumura N Mandai M . Dynamic host immunity and PD-L1/PD-1 blockade efficacy: developments after “IFN-γ from lymphocytes induces PD-L1 expression and promotes progression of ovarian cancer”. Br J Cancer (2022). doi: 10.1038/s41416-022-01960-x PMC9938281 36068276

[60] Padmanabhan S Gaire B de Leon D Vancura A Vancurova I . Interferon-γ induces PD-L1 expression in ovarian cancer cells by JAK/STAT1 signaling. FASEB J (2020) 34:1–1. doi: 10.1096/fasebj.2020.34.s1.01874

[61] Padmanabhan S Gaire B Zou Y Uddin MM Vancurova I . IFNγ-induced PD-L1 expression in ovarian cancer cells is regulated by JAK1, STAT1 and IRF1 signaling. Cell Signalling (2022) 97:110400. doi: 10.1016/j.cellsig.2022.110400 35820543 PMC9357219

[62] Padmanabhan S Gaire B Vancura A Vancurova I . Interferon-γ induced PD-L1 expression in ovarian cancer cells is regulated by IRF1 signaling. FASEB J (2022) 97:110400. doi: 10.1096/fasebj.2022.36.S1.R3152 PMC9357219 35820543

[63] Padmanabhan S Zou Y Vancurova I . Immunoblotting analysis of intracellular PD-L1 levels in interferon-γ-Treated ovarian cancer cells stably transfected with Bcl3 shRNA. In: : Vancurova I Zhu Y , editors. Immune mediators in cancer: Methods and protocols. New York, NY: Springer US (2020). p. p211–220. doi: 10.1007/978-1-0716-0247-8_18 31939183

[64] Zou Y Uddin MM Padmanabhan S Zhu Y Bu P Vancura A . The proto-oncogene Bcl3 induces immune checkpoint PD-L1 expression, mediating proliferation of ovarian cancer cells. J Biol Chem (2018) 293:15483–96. doi: 10.1074/jbc.RA118.004084 PMC6177577 30135206

[65] Ni Y Soliman A Joehlin-Price A Abdul-Karim F Rose PG Mahdi H . Immune cells and signatures characterize tumor microenvironment and predict outcome in ovarian and endometrial cancers. Immunotherapy (2021) 13:1179–92. doi: 10.2217/imt-2021-0052 34424031

[66] Bruand M Barras D Mina M Lanitis E Chong C Dorier J . Immunogenicity of BRCA1-deficient ovarian cancers is driven through DNA sensing and is augmented by PARP inhibition. Ann Oncol (2019) 30:v761. doi: 10.1093/annonc/mdz268.003

[67] Hernando JJ Park T-W Fischer H-P Zivanovic O Braun M Pölcher M . Vaccination with dendritic cells transfected with mRNA-encoded folate-receptor-α for relapsed metastatic ovarian cancer. Lancet Oncol (2007) 8:451–4. doi: 10.1016/S1470-2045(07)70142-0 17466904

[68] Adhikary T Wortmann A Finkernagel F Lieber S Nist A Stiewe T . Interferon signaling in ascites-associated macrophages is linked to a favorable clinical outcome in a subgroup of ovarian carcinoma patients. BMC Genomics (2017) 18:243. doi: 10.1186/s12864-017-3630-9 28327095 PMC5359932

[69] Dangaj D Bruand M Grimm AJ Ronet C Barras D Duttagupta PA . Cooperation between constitutive and inducible chemokines enables T cell engraftment and immune attack in solid tumors. Cancer Cell (2019) 35:885–900.e10. doi: 10.1016/j.ccell.2019.05.004 31185212 PMC6961655

[70] Mohamed E Sierra RA Trillo-Tinoco J Cao Y Innamarato P Payne KK . The unfolded protein response mediator PERK governs myeloid cell-driven immunosuppression in tumors through inhibition of STING signaling. Immunity (2020) 52:668–682.e7. doi: 10.1016/j.immuni.2020.03.004 32294407 PMC7207019

[71] Lisci M Barton PR Randzavola LO Ma CY Marchingo JM Cantrell DA . Mitochondrial translation is required for sustained killing by cytotoxic T cells. Science (2021) 374:eabe9977. doi: 10.1126/science.abe9977 34648346

[72] Li Y Yang Y Xiong A Wu X Xie J Han S . Comparative gene expression analysis of lymphocytes treated with exosomes derived from ovarian cancer and ovarian cysts. Front Immunol (2017) 8:607. doi: 10.3389/fimmu.2017.00607 28620375 PMC5451634

[73] McLaughlin M Patin EC Pedersen M Wilkins A Dillon MT Melcher AA . Inflammatory microenvironment remodelling by tumour cells after radiotherapy. Nat Rev Cancer (2020) 20:203–17. doi: 10.1038/s41568-020-0246-1 32161398

[74] Kho VM Mekers VE Span PN Bussink J Adema GJ . Radiotherapy and cGAS/STING signaling: Impact on MDSCs in the tumor microenvironment. Cell Immunol (2021) 362:104298. doi: 10.1016/j.cellimm.2021.104298 33592541

[75] Yum S Li M Chen ZJ . Old dogs, new trick: classic cancer therapies activate cGAS. Cell Res (2020) 30:639–48. doi: 10.1038/s41422-020-0346-1 PMC7395767 32541866

[76] Burnette BC Liang H Lee Y Chlewicki L Khodarev NN Weichselbaum RR . The efficacy of radiotherapy relies upon induction of type I interferon–dependent innate and adaptive immunity. Cancer Res (2011) 71:2488–96. doi: 10.1158/0008-5472.CAN-10-2820 PMC3070872 21300764

[77] Deng L Liang H Xu M Yang X Burnette B Arina A . STING-dependent cytosolic DNA sensing promotes radiation-induced type I interferon-dependent antitumor immunity in immunogenic tumors. Immunity (2014) 41:843–52. doi: 10.1016/j.immuni.2014.10.019 PMC5155593 25517616

[78] Zheng W Ranoa DRE Huang X Hou Y Yang K Poli EC . RIG-I–like receptor LGP2 is required for tumor control by radiotherapy. Cancer Res (2020) 80:5633–41. doi: 10.1158/0008-5472.CAN-20-2324 33087322

[79] De Martino M Daviaud C Vanpouille-Box C . Radiotherapy: An immune response modifier for immuno-oncology. Semin Immunol (2021) 52:101474. doi: 10.1016/j.smim.2021.101474 33741223

[80] Vanpouille-Box C Alard A Aryankalayil MJ Sarfraz Y Diamond JM Schneider RJ . DNA Exonuclease Trex1 regulates radiotherapy-induced tumour immunogenicity. Nat Commun (2017) 8:15618. doi: 10.1038/ncomms15618 28598415 PMC5472757

[81] Gregg RW Sarkar SN Shoemaker JE . Mathematical modeling of the cGAS pathway reveals robustness of DNA sensing to TREX1 feedback. J Theor Biol (2019) 462:148–57. doi: 10.1016/j.jtbi.2018.11.001 30395807

[82] Lugade AA Sorensen EW Gerber SA Moran JP Frelinger JG Lord EM . Radiation-induced IFN-γ production within the tumor microenvironment influences antitumor immunity. J Immunol (2008) 180:3132–9. doi: 10.4049/jimmunol.180.5.3132 18292536

[83] Gerber SA Sedlacek AL Cron KR Murphy SP Frelinger JG Lord EM . IFN-γ mediates the antitumor effects of radiation therapy in a murine colon tumor. Am J Pathol (2013) 182:2345–54. doi: 10.1016/j.ajpath.2013.02.041 PMC3668027 23583648

[84] Herrera FG Irving M Kandalaft LE Coukos G . Rational combinations of immunotherapy with radiotherapy in ovarian cancer. Lancet Oncol (2019) 20:e417–33. doi: 10.1016/S1470-2045(19)30401-2 31364594

[85] He K Patel RR Barsoumian HB Chang JY Tang C Comeaux NI . Phase II trial of high-dose radiotherapy vs. low-dose radiation, demonstrating low-dose mediated immune-cell infiltration. Int J Radiat OncologyBiologyPhysics (2021) 111:S118. doi: 10.1016/j.ijrobp.2021.07.270

[86] Herrera FG Ronet C Ochoa de Olza M Barras D Crespo I Andreatta M . Low-dose radiotherapy reverses tumor immune desertification and resistance to immunotherapy. Cancer Discovery (2022) 12:108–33. doi: 10.1158/2159-8290.CD-21-0003 PMC9401506 34479871

[87] Wang W Kryczek I Dostál L Lin H Tan L Zhao L . Effector T cells abrogate stroma-mediated chemoresistance in ovarian cancer. Cell (2016) 165:1092–105. doi: 10.1016/j.cell.2016.04.009 PMC4874853 27133165

[88] Zhang Q Wang J Qiao H Huyan L Liu B Li C . ISG15 is downregulated by KLF12 and implicated in maintenance of cancer stem cell-like features in cisplatin-resistant ovarian cancer. J Cell Mol Med (2021) 25:4395–407. doi: 10.1111/jcmm.16503 PMC8093991 33797839

[89] Acland M Lokman NA Young C Anderson D Condina M Desire C . Chemoresistant cancer cell lines are characterized by migratory, amino acid metabolism, protein catabolism and IFN1 signalling perturbations. Cancers (Basel) (2022) 14:2763. doi: 10.3390/cancers14112763 35681748 PMC9179525

[90] Li Y Wang H Chen M Ma X . The immune subtype contributes to distinct overall survival for ovarian cancer patients with platinum-based adjuvant therapy. Front Immunol (2022) 13:872991. doi: 10.3389/fimmu.2022.872991 35812434 PMC9263722

[91] Yang P-M Hsieh Y-Y Du J-L Yen S-C Hung C-F . Sequential interferon β-cisplatin treatment enhances the surface exposure of calreticulin in cancer cells *via* an interferon regulatory factor 1-dependent manner. Biomolecules (2020) 10:643. doi: 10.3390/biom10040643 32326356 PMC7226424

[92] Borucka J Sterzyńska K Kaźmierczak D Świerczewska M Nowacka M Wojtowicz K . The significance of interferon gamma inducible protein 16 (IFI16) expression in drug resistant ovarian cancer cell lines. Biomedicine Pharmacother (2022) 150:113036. doi: 10.1016/j.biopha.2022.113036 35489285

[93] Galluzzi L Humeau J Buqué A Zitvogel L Kroemer G . Immunostimulation with chemotherapy in the era of immune checkpoint inhibitors. Nat Rev Clin Oncol (2020) 17:725–41. doi: 10.1038/s41571-020-0413-z 32760014

[94] Ashworth A Lord CJ . Synthetic lethal therapies for cancer: what’s next after PARP inhibitors? Nat Rev Clin Oncol (2018) 15:564–76. doi: 10.1038/s41571-018-0055-6 29955114

[95] Ding L Kim H-J Wang Q Kearns M Jiang T Ohlson CE . PARP inhibition elicits STING-dependent antitumor immunity in Brca1-deficient ovarian cancer. Cell Rep (2018) 25:2972–2980.e5. doi: 10.1016/j.celrep.2018.11.054 30540933 PMC6366450

[96] Shen J Zhao W Ju Z Wang L Peng Y Labrie M . PARPi triggers the STING-dependent immune response and enhances the therapeutic efficacy of immune checkpoint blockade independent of BRCAness. Cancer Res (2019) 79:311–9. doi: 10.1158/0008-5472.CAN-18-1003 PMC6588002 30482774

[97] Martincuks A Song J Kohut A Zhang C Li Y-J Zhao Q . PARP inhibition activates STAT3 in both tumor and immune cells underlying therapy resistance and immunosuppression in ovarian cancer. Front Oncol (2021) 11:724104. doi: 10.3389/fonc.2021.724104 34956861 PMC8693573

[98] Mehdipour P Marhon SA Ettayebi I Chakravarthy A Hosseini A Wang Y . Epigenetic therapy induces transcription of inverted SINEs and ADAR1 dependency. Nature (2020) 588:169–73. doi: 10.1038/s41586-020-2844-1 33087935

[99] McDonald JI Diab N Arthofer E Hadley M Kanholm T Rentia U . Epigenetic therapies in ovarian cancer alter repetitive element expression in a *TP53* - dependent manner. Cancer Res (2021) 81:5176–89. doi: 10.1158/0008-5472.CAN-20-4243 PMC8530980 34433584

[100] Stone ML Chiappinelli KB Li H Murphy LM Travers ME Topper MJ . Epigenetic therapy activates type I interferon signaling in murine ovarian cancer to reduce immunosuppression and tumor burden. Proc Natl Acad Sci U.S.A. (2017) 114:E10981–90. doi: 10.1073/pnas.1712514114 PMC5754782 29203668

[101] Arthofer E Diab N Chiappinelli KB . Abstract B20: p53 regulation of repetitive elements and the interferon response in cancer. Cancer Immunol Res (2020) 8:B20–0. doi: 10.1158/2326-6074.TUMIMM18-B20

[102] Moufarrij S Srivastava A Gomez S Hadley M Palmer E Austin PT . Combining DNMT and HDAC6 inhibitors increases anti-tumor immune signaling and decreases tumor burden in ovarian cancer. Sci Rep (2020) 10:3470. doi: 10.1038/s41598-020-60409-4 32103105 PMC7044433

[103] Soldi R Ghosh Halder T Weston A Thode T Drenner K Lewis R . The novel reversible LSD1 inhibitor SP-2577 promotes anti-tumor immunity in SWItch/Sucrose-NonFermentable (SWI/SNF) complex mutated ovarian cancer. PloS One (2020) 15:e0235705. doi: 10.1371/journal.pone.0235705 32649682 PMC7351179

[104] Cornelison R Biswas K Llaneza DC Harris AR Sosale NG Lazzara MJ . CX-5461 treatment leads to cytosolic DNA-mediated STING activation in ovarian cancer. Cancers (Basel) (2021) 13:5056. doi: 10.3390/cancers13205056 34680204 PMC8533980

[105] Waddell C Price M Johnson P Edmondson R Owens G . P06.10 short term inhibition of checkpoint proteins increases ex vivo expansion of tumour infiltrating lymphocytes in high grade serous ovarian cancer. J Immunother Cancer (2020) 8:A45. doi: 10.1136/jitc-2020-ITOC7.89

[106] Garris CS Arlauckas SP Kohler RH Trefny MP Garren S Piot C . Successful anti-PD-1 cancer immunotherapy requires T cell-dendritic cell crosstalk involving the cytokines IFN-γ and IL-12. Immunity (2018) 49:1148–1161.e7. doi: 10.1016/j.immuni.2018.09.024 30552023 PMC6301092

[107] Chen X Pan X Zhang W Guo H Cheng S He Q . Epigenetic strategies synergize with PD-L1/PD-1 targeted cancer immunotherapies to enhance antitumor responses. Acta Pharm Sin B (2020) 10:723–33. doi: 10.1016/j.apsb.2019.09.006 PMC7276686 32528824

[108] Shakfa N Lightbody E Li D Wilson-Sanchez J Conseil G Afriyie-Asante A . Abstract 1708: Improving genotype specific chemotherapy response in ovarian cancer *via* cGAS-STING pathway activation. Cancer Res (2021) 81:1708–8. doi: 10.1158/1538-7445.AM2021-1708

[109] Yahata T Mizoguchi M Kimura A Orimo T Toujima S Kuninaka Y . Programmed cell death ligand 1 disruption by clustered regularly interspaced short palindromic repeats/Cas9-genome editing promotes antitumor immunity and suppresses ovarian cancer progression. Cancer Sci (2019) 110:1279–92. doi: 10.1111/cas.13958 PMC6447841 30702189

[110] Felices M Chu S Kodal B Bendzick L Ryan C Lenvik AJ . IL-15 super-agonist (ALT-803) enhances natural killer (NK) cell function against ovarian cancer. Gynecologic Oncol (2017) 145:453–61. doi: 10.1016/j.ygyno.2017.02.028 PMC5447472 28236454

[111] Zeng Y Li B Liang Y Reeves PM Qu X Ran C . Dual blockade of CXCL12-CXCR4 and PD-1–PD-L1 pathways prolongs survival of ovarian tumor–bearing mice by prevention of immunosuppression in the tumor microenvironment. FASEB J (2019) 33:6596–608. doi: 10.1096/fj.201802067RR PMC6463916 30802149

[112] Zhang Q-F Li J Jiang K Wang R Ge J Yang H . CDK4/6 inhibition promotes immune infiltration in ovarian cancer and synergizes with PD-1 blockade in a b cell-dependent manner. Theranostics (2020) 10:10619–33. doi: 10.7150/thno.44871 PMC7482823 32929370

[113] Kalbasi A Ribas A . Tumour-intrinsic resistance to immune checkpoint blockade. Nat Rev Immunol (2020) 20:25–39. doi: 10.1038/s41577-019-0218-4 31570880 PMC8499690

[114] Shin DS Zaretsky JM Escuin-Ordinas H Garcia-Diaz A Hu-Lieskovan S Kalbasi A . Primary resistance to PD-1 blockade mediated by JAK1/2 mutations. Cancer Discovery (2017) 7:188–201. doi: 10.1158/2159-8290.CD-16-1223 27903500 PMC5296316

[115] Liu J Ma J Xing N Ji Z Li J Zhang S . Interferon-γ predicts the treatment efficiency of immune checkpoint inhibitors in cancer patients. J Cancer Res Clin Oncol (2022). doi: 10.1007/s00432-022-04201-z PMC11796581 35852620

[116] Liu C He H Li X Su MA Cao Y . Dynamic metrics-based biomarkers to predict responders to anti-PD-1 immunotherapy. Br J Cancer (2019) 120:346–55. doi: 10.1038/s41416-018-0363-8 PMC6353899 30587849

[117] Xing P Zhang J Liu R Wang J Ma M Wang L . IFN-α, IFN-γ, IL-2 combined with TNF-α for predicting efficacy of PD-1 inhibitors combination therapy in patients with solid cancers. JCO (2021) 39:2584–4. doi: 10.1200/JCO.2021.39.15_suppl.2584

[118] Van der Meer JMR Maas RJA Guldevall K Klarenaar K de Jonge PKJD Evert JSH . IL-15 superagonist n-803 improves IFNγ production and killing of leukemia and ovarian cancer cells by CD34^+^ progenitor-derived NK cells. Cancer Immunol Immunother (2021) 70:1305–21. doi: 10.1007/s00262-020-02749-8 PMC8053152 33140189

[119] Liu C Huang Y Cui Y Zhou J Qin X Zhang L . The immunological role of CDK4/6 and potential mechanism exploration in ovarian cancer. Front Immunol (2022) 12:799171. doi: 10.3389/fimmu.2021.799171 35095879 PMC8795791

[120] Deng C Zhao J Zhou S Dong J Cao J Gao J . The vascular disrupting agent CA4P improves the antitumor efficacy of CAR-T cells in preclinical models of solid human tumors. Mol Ther (2020) 28:75–88. doi: 10.1016/j.ymthe.2019.10.010 31672285 PMC6953963

[121] Boulch M Cazaux M Loe-Mie Y Thibaut R Corre B Lemaître F . A cross-talk between CAR T cell subsets and the tumor microenvironment is essential for sustained cytotoxic activity. Sci Immunol (2021) 6(57):eabd4344. doi: 10.1126/sciimmunol.abd4344 33771887

[122] Owens GL Sheard VE Kalaitsidou M Blount D Lad Y Cheadle EJ . Preclinical assessment of CAR T-cell therapy targeting the tumor antigen 5T4 in ovarian cancer. J Immunother (2018) 41:130–40. doi: 10.1097/CJI.0000000000000203 PMC5895166 29239915

[123] Guo C Dong E Lai Q Zhou S Zhang G Wu M . Effective antitumor activity of 5T4-specific CAR-T cells against ovarian cancer cells *in vitro* and xenotransplanted tumors *in vivo* . MedComm (2020) (2020) 1:338–50. doi: 10.1002/mco2.34 PMC8491242 34766126

[124] Dobrzanski MJ Rewers-Felkins KA Samad KA Quinlin IS Phillips CA Robinson W . Immunotherapy with IL-10- and IFN-γ-producing CD4 effector cells modulate “Natural” and “Inducible” CD4 TReg cell subpopulation levels: observations in four cases of patients with ovarian cancer. Cancer Immunol Immunother (2012) 61:839–54. doi: 10.1007/s00262-011-1128-x PMC3315597 22083345

[125] Li T Wang J . Therapeutic effect of dual CAR-T targeting PDL1 and MUC16 antigens on ovarian cancer cells in mice. BMC Cancer (2020) 20:678. doi: 10.1186/s12885-020-07180-x 32689954 PMC7372885

[126] Bailey S Vatsa S Bouffard A Larson R Scarfo I Kann M . 767 interferon gamma reduces CAR-T exhaustion and toxicity without compromising therapeutic efficacy in hematologic malignancies. In: Late-breaking abstracts. BMJ Publishing Group Ltd (2020). p. A459. doi: 10.1136/jitc-2020-SITC2020.0767

[127] Castiello L Sabatino M Jin P Clayberger C Marincola FM Krensky AM . Monocyte-derived DC maturation strategies and related pathways: a transcriptional view. Cancer Immunol Immunother (2011) . 60(4):457–66. doi: 10.1007/s00262-010-0954-6 PMC3086891 21258790

[128] Tanyi JL Bobisse S Ophir E Tuyaerts S Roberti A Genolet R . Personalized cancer vaccine effectively mobilizes antitumor T cell immunity in ovarian cancer. Sci Transl Med (2018) 10:eaao5931. doi: 10.1126/scitranslmed.aao5931 29643231

[129] Klapdor R Shuo W Morgan MA Zimmermann K Hachenberg J Büning H . NK cell-mediated eradication of ovarian cancer cells with a novel chimeric antigen receptor directed against CD44. NK cell-mediated eradication of ovarian cancer cells with a novel chimeric antigen receptor directed against CD44. Biomedicines (2021) 9:1339. doi: 10.3390/biomedicines9101339 PMC8533227 34680456

[130] Klapdor R Wang S Hacker U Büning H Morgan M Dörk T . Improved killing of ovarian cancer stem cells by combining a novel chimeric antigen receptor–based immunotherapy and chemotherapy. Hum Gene Ther (2017) 28:886–96. doi: 10.1089/hum.2017.168 28836469

[131] Dold C Rodriguez Urbiola C Wollmann G Egerer L Muik A Bellmann L . Application of interferon modulators to overcome partial resistance of human ovarian cancers to VSV-GP oncolytic viral therapy. Mol Ther - Oncolytics (2016) 3:16021. doi: 10.1038/mto.2016.21 27738655 PMC5040171

[132] Santos JM Heiniö C Cervera-Carrascon V Quixabeira DCA Siurala M Havunen R . Oncolytic adenovirus shapes the ovarian tumor microenvironment for potent tumor-infiltrating lymphocyte tumor reactivity. J Immunother Cancer (2020) 8:e000188. doi: 10.1136/jitc-2019-000188 31940588 PMC7057530

[133] de Queiroz NMGP Xia T Konno H Barber GN . Ovarian cancer cells commonly exhibit defective STING signaling which affects sensitivity to viral oncolysis. Mol Cancer Res (2019) 17:974–86. doi: 10.1158/1541-7786.MCR-18-0504 PMC6445711 30587523

[134] Matveeva OV Chumakov PM . Defects in interferon pathways as potential biomarkers of sensitivity to oncolytic viruses. Rev Med Virol (2018) 28(6):e2008. doi: 10.1002/rmv.2008 30209859 PMC6906582

[135] Delaunay T Achard C Boisgerault N Grard M Petithomme T Chatelain C . Frequent homozygous deletions of type I interferon genes in pleural mesothelioma confer sensitivity to oncolytic measles virus. J Thorac Oncol (2020) 15(5):827–42. doi: 10.1016/j.jtho.2019.12.128 31945495

[136] Nguyen TT Ramsay L Ahanfeshar-Adams M Lajoie M Schadendorf D Alain T . Mutations in the IFNγ-JAK-STAT pathway causing resistance to immune checkpoint inhibitors in melanoma increase sensitivity to oncolytic virus treatment. Clin Cancer Res (2021) 27(12):3432–42. doi: 10.1158/1078-0432.CCR-20-3365 33593882

[137] Schuster P Lindner G Thomann S Haferkamp S Schmidt B . Prospect of plasmacytoid dendritic cells in enhancing anti-tumor immunity of oncolytic herpes viruses. Cancers (2019) 11:651. doi: 10.3390/cancers11050651 31083559 PMC6562787

[138] Montagner IM Merlo A Carpanese D Dalla Pietà A Mero A Grigoletto A . A site-selective hyaluronan-interferonα2a conjugate for the treatment of ovarian cancer. J Controlled Release (2016) 236:79–89. doi: 10.1016/j.jconrel.2016.06.033 27356018

[139] Iwamura T Narumi H Suzuki T Yanai H Mori K Yamashita K . Novel pegylated interferon-β as strong suppressor of the malignant ascites in a peritoneal metastasis model of human cancer. Cancer Sci (2017) 108:581–9. doi: 10.1111/cas.13176 PMC5406538 28129467

[140] Agarwal Y Milling LE Chang JYH Santollani L Sheen A Lutz EA . Intratumourally injected alum-tethered cytokines elicit potent and safer local and systemic anticancer immunity. Nat BioMed Eng (2022) 6:129–43. doi: 10.1038/s41551-021-00831-9 PMC9681025 35013574

[141] Lutz EA Agarwal Y Momin N Cowles SC Palmeri JR Duong E . Alum-anchored intratumoral retention improves the tolerability and antitumor efficacy of type I interferon therapies. Proc Natl Acad Sci (2022) 119:e2205983119. doi: 10.1073/pnas.2205983119 36037341 PMC9457244

[142] Imamura Y Tashiro H Tsend-Ayush G Haruta M Dashdemberel N Komohara Y . Novel therapeutic strategies for advanced ovarian cancer by using induced pluripotent stem cell-derived myelomonocytic cells producing interferon beta. Cancer Sci (2018) 109:3403–10. doi: 10.1111/cas.13775 PMC6215869 30142694

[143] Sun L Kees T Almeida AS Liu B He X-Y Ng D . Activating a collaborative innate-adaptive immune response to control metastasis. Cancer Cell (2021) 39:1361–74.e9. doi: 10.1016/j.ccell.2021.08.005 34478639 PMC8981964

[144] Green DS Husain SR Johnson CL Sato Y Han J Joshi B . Combination immunotherapy with IL-4 *Pseudomonas* exotoxin and IFN-α and IFN-γ mediate antitumor effects *in vitro* and in a mouse model of human ovarian cancer. Immunotherapy (2019) 11:483–96. doi: 10.2217/imt-2018-0158 PMC6439502 30860437

[145] Green DS Nunes AT David-Ocampo V Ekwede IB Houston ND Highfill SL . A phase 1 trial of autologous monocytes stimulated ex vivo with sylatron® (Peginterferon alfa-2b) and actimmune® (Interferon gamma-1b) for intra-peritoneal administration in recurrent ovarian cancer. J Transl Med (2018) 16:196. doi: 10.1186/s12967-018-1569-5 30012146 PMC6048715

[146] Green DS Nunes AT Tosh KW David-Ocampo V Fellowes VS Ren J . Production of a cellular product consisting of monocytes stimulated with sylatron® (Peginterferon alfa-2b) and actimmune® (Interferon gamma-1b) for human use. J Transl Med (2019) 17:82. doi: 10.1186/s12967-019-1822-6 30871636 PMC6419352

[147] Mistarz A Graczyk M Winkler M Singh PK Cortes E Miliotto A . Induction of cell death in ovarian cancer cells by doxorubicin and oncolytic vaccinia virus is associated with CREB3L1 activation. Mol Ther - Oncolytics (2021) 23:38–50. doi: 10.1016/j.omto.2021.04.014 34632049 PMC8479291

[148] Yi R Lv W Zheng S Zhang N Zhang Y Yang K . IFN-γ/Doxorubicin complex nanoparticles for enhancing therapy in the context of human ovarian carcinoma. Front Mater (2022) 9:944930. doi: 10.3389/fmats.2022.944930

[149] Gozgit JM Vasbinder MM Abo RP Kunii K Kuplast-Barr KG Gui B . PARP7 negatively regulates the type I interferon response in cancer cells and its inhibition triggers antitumor immunity. Cancer Cell (2021) 39:1214–26.e10. doi: 10.1016/j.ccell.2021.06.018 34375612

[150] Falchook GS Patel MR Yap TA McEachern K Kuplast-Barr K Utley L . A first-in-human phase 1 study of a novel PARP7 inhibitor RBN-2397 in patients with advanced solid tumors. JCO (2021) 39:3000–0. doi: 10.1200/JCO.2021.39.15_suppl.3000

[151] Li X Luo L Jiang M Zhu C Shi Y Zhang J . Cocktail strategy for ‘cold’ tumors therapy *via* active recruitment of CD8^+^ T cells and enhancing their function. J Controlled Release (2021) 334:413–26. doi: 10.1016/j.jconrel.2021.05.002 33964366

